# Skeletal muscle atrophy and dysfunction in obesity and type-2 diabetes mellitus: Myocellular mechanisms involved

**DOI:** 10.1007/s11154-025-09954-9

**Published:** 2025-03-10

**Authors:** Íñigo M. Pérez Castillo, Josep M. Argilés, Ricardo Rueda, María Ramírez, José M. López Pedrosa

**Affiliations:** 1 Abbott Nutrition R&D, Abbott Laboratories, 18004 Granada, Spain; 2 Cancer Research Group, Departament de Bioquímica I Molecular Biomedicine, Facultat de Biologia, Barcelona, Spain and Institut de Biomedicina de La Barcelona (IBUB), Universitat de Barcelona, Barcelona, Spain

**Keywords:** Obesity, Type 2 diabetes mellitus, Skeletal muscle, Atrophy, Dysfunction, Mechanisms

## Abstract

Obesity and type-2 diabetes mellitus (T2DM) are interrelated metabolic disorders primarily driven by overnutrition and physical inactivity, which oftentimes entails a transition from obesity to T2DM. Compromised musculoskeletal health consistently emerges as a common hallmark in the progression of these metabolic disorders. Skeletal muscle atrophy and dysfunction can further impair whole-body metabolism and reduce physical exercise capacity, thus instigating a vicious cycle that further deteriorates the underlying conditions. However, the myocellular repercussions of these metabolic disturbances remain to be completely clarified. Insulin signaling not only facilitates skeletal muscle glucose uptake but also plays a central role in skeletal muscle anabolism mainly due to suppression of catabolic pathways and facilitating an anabolic response to nutrient feeding. Chronic overnutrition may trigger different myocellular mechanisms proposed to contribute to insulin resistance and aggravate skeletal muscle atrophy and dysfunction. These mechanisms mainly include the inactivation of insulin signaling components through sustained activation of stress-related pathways, mitochondrial dysfunction, a shift to glycolytic skeletal muscle fibers, and hyperglycemia. In the present review, we aim to delve on these mechanisms, providing an overview of the myocellular processes involved in skeletal muscle atrophy and dysfunction under chronic overnutrition, and their contribution to the progression to T2DM.

## Introduction

Obesity and type-2 diabetes mellitus (T2DM) are closely related clinical conditions that have reached pandemic proportions, significantly overlapping in several factors responsible for their etiology, pathogenesis, and treatment [1]. In 2022, the global age-standardized prevalence of diabetes assessed based on fasting glucose, glycated hemoglobin or use of hypoglycemic drugs or insulin was 13.9% for women and 14.3% for men, accounting for 828 million adults (T2DM accounts for 85–95% of diabetes cases in adults) [2]. This represents an increase of 630 million adults compared to 1990 data. Estimates of age-standardized diabetes prevalence are notably higher, even surpassing 25%, in low- and middle-income countries in Polynesia and Micronesia, Middle East and North of Africa compared to the lowest estimates of 2–4% reported in European countries such as France, Denmark, and Spain [2]. Similarly, the global age-standardized prevalence of obesity, defined as a body mass index (BMI) ≥ 30 (used as an index of adiposity due to practical considerations), increased from 8.8% to 18.5% in women, and from 4.8% to 14.0% in men during the same time period. This represents an estimated increase of 684 million adults [3]. Low- and middle-income countries in Polynesia and Micronesia, Middle East and North of Africa have experienced the largest increments in prevalence of obesity in adults, whereas some European countries (e.g., Spain and France) have seen flattened or decreased prevalence during the same time period [3]. These data reinforce comprehensive longitudinal evidence positioning obesity as a major risk factor in the onset of T2DM [4, 5]. A recent systematic analysis of 216 cohort studies involving 26 million individuals concluded that each 5-unit increase in BMI associates with a 72% higher risk of T2DM [6]. This risk intensifies with increased ectopic or visceral fat content at any BMI level [6, 7]. Consequently, coexistence of both conditions, oftentimes termed “diabesity” [8], is a reality for many individuals, and it typically entails a transition from obesity to T2DM. To prevent T2DM progression, the critical role of body composition management is nowadays well-recognized, which is typically achieved through lifestyle interventions including physical activity programs and nutritional strategies as well as adjunct pharmacotherapy and surgery when needed [1].

The skeletal muscle represents ≈40% of total body weight under physiological conditions and contains 50–75% of all body proteins [9]. It serves as the primary reservoir of glucose (stored as glycogen) [10], accounts for ≈80% glucose disposal under hyperinsulinemic-euglycemic conditions [11], and is responsible for 30% to over 90% of whole-body metabolic rate at rest and during intense physical exercise, respectively [12]. Further, the skeletal muscle is described as the largest secretory organ in the normal-weight human body, exerting autocrine, paracrine and endocrine effects implicated in metabolic homeostasis [13]. As such, a growing body of evidence focuses on impaired skeletal muscle plasticity – changes in muscle mass, and quality/function to meet metabolic requirements – as a key player in the onset and progression of metabolic disorders such as obesity and T2DM [14, 15].

Although not all obese individuals exhibit insulin resistance – a condition that some authors referred to as ‘metabolically healthy obesity’ [16] – and patients with T2DM are not invariably obese [17], compromised musculoskeletal health consistently emerges as a common hallmark in both conditions. T2DM patients display skeletal muscle atrophy denoted by greater loss of muscle mass compared to age-matched subjects without diabetes [18, 19]. Specifically, the decline in skeletal muscle mass associated with aging has been reported to occur at twice the rate in individuals with T2DM compared to non-diabetic older adults [18]. Compared to non-diabetic matched controls, T2DM patients also display reduced absolute skeletal muscle strength [20]. Individuals with diabetes have approximately 1.5 times the odds of developing sarcopenia, as determined by criteria that include both muscle mass and function parameters, compared to non-diabetic subjects [21]. Unlike diabetes, obesity per se is not typically associated with lower absolute skeletal muscle mass [22, 23]. In fact, increased adiposity has been observed to correlate with higher absolute measurements of muscle strength [24]. This phenomenon is frequently explained by the augmented mechanical loading stimulus that skeletal muscles experience due to higher body weight, especially those responsible for maintaining posture [25]. However, when normalized to total mass, subjects with obesity do exhibit impaired skeletal muscle function (i.e., lower strength measurements relative to muscle mass) [24, 26], thus indicating reduced muscle quality. Further, a shift from highly-vascularized oxidative type-I fibers to glycolytic type-IIx fibers has been reported in obese, and diabetic skeletal muscles [27–33], which might correlate with increased fatigability, albeit observations are not always consistent [34]. Along with macrovascular complications, T2DM associates with capillary rarefaction [35], altered microvascular network structure [36], and impaired endovascular function [35], altogether compromising blood flow to the skeletal muscle. Signs of impaired microvasculature have been also documented in patients with obesity, which are typically linked to the onset of insulin resistance [37, 38]. Further, lipid oversupply in obesity leads to fat redistribution to ectopic locations, including non-adipose tissues such as the skeletal muscle, where lipid droplets accumulate to the subsarcolemmal region or in-between myofibrils [39]. Co-existence of obesity and T2DM associates with presence of remarkably enlarged lipid droplets, which might locate preferentially to type-II myofibers [40]. While a similar phenomenon of increased intramyocellular lipid (IMCL) content is known to occur in endurance-trained athletes, the preferential distribution to type-I myofibers, and the lower lipid droplet size suggest that these may serve a physiological purpose of facilitating efficient energy supply through β-oxidation during long-duration exercise in metabolically healthy individuals [41]. On the other hand, IMCL accumulation in obesity and T2DM leads to a loss of muscle contractile properties, and deleterious metabolic effects termed “lipotoxicity” [42, 43]. These, compounded with other histological features of obese and diabetic muscles (e.g., expanded extracellular matrix (ECM) component [44, 45]), are thought to contribute to impaired skeletal muscle function and decreased exercise tolerance [46].

It is worth noting that differences in skeletal muscle biology have been also reported between metabolically healthy and unhealthy obese phenotypes [47], yet differences between these groups of study strongly rely on the criteria used to classify them. Besides metabolic syndrome components, BMI may be insufficient to match metabolically healthy and unhealthy obese subjects in analyses and different body composition parameters have been suggested to contribute to metabolic health. Particularly, metabolically unhealthy individuals have been reported to present similar percentage body fat adjusted by BMI and sex, but increased intra-abdominal adipose tissue and intra-hepatic triglyceride content than those classified as metabolically healthy [48]. Non-invasive body composition assessment techniques, such as magnetic resonance imaging (MRI) and spectroscopy (MRS) may support assessments of regional body fat distribution in these individuals and evaluate its potential impact on muscle health [49]. Metabolically healthy obesity remains a topic of scientific debate, and approaches to characterize these phenotypes have been reviewed elsewhere [50].

Abovementioned deleterious effects of obesity and T2DM on skeletal muscle have significant implications for its metabolic and anabolic properties. In turn, these impairments contribute to a loss of functionality and compromised ability to perform physical activity and exercise, thus representing a vicious cycle that reinforces the underlying metabolic conditions [51]. However, the cellular and molecular mechanisms underlying the phenotypical changes of the skeletal muscle induced by these metabolic disarrangements remain unclear. The goal of the present narrative review is to provide an overview of the myocellular mechanisms involved in the loss of skeletal muscle mass and function associated with obesity and T2DM, deepening our understanding of skeletal muscle atrophy and dysfunction as both potential cause and consequence of these metabolic conditions.

## Metabolic signatures of obesity and T2DM implicated in musculoskeletal health

Inflammation, impaired adipocyte endocrine function, and ectopic tissue lipotoxicity have all been proposed as causes of insulin resistance (IR), and consequently T2DM in obese individuals. However, efforts to pinpoint a cause-effect relationship, elucidate initializing factors, or clarify whether IR propagates from one initial tissue to others have been shown largely unsuccessful [52]. Notwithstanding, a growing body of evidence supports the notion of adipocyte hypertrophy (Box 1) as an early contributor to metabolic dysfunction and the onset of systemic IR [53, 54]. For instance, plasma levels of adiponectin – a potent anti-inflammatory adipokine with triglyceride- and glucose-lowering effects on skeletal muscle, whose production is dysregulated in hypertrophic/fibrotic adipocytes [55, 56] – indirectly correlate with IR [57]. Landmark preclinical research demonstrated that overexpression of tumor necrosis factor α (TNF-α) induced in obese adipose tissue impairs peripheral glucose uptake in response to insulin [58]. There is compelling evidence of exacerbated renin-angiotensin (RAS) components expression in visceral adipose tissue in obese individuals, which correlates with increased circulating angiotensin II levels and different metabolic complications, including IR [59]. Several different adipokines (i.e., leptin, interleukin-6 (IL-6), IL-1β, etc.) have been involved in mechanisms causative of IR; however, some authors have questioned whether local increases in adipose tissue translate to systemic concentrations that are sufficient to impair systemic insulin sensitivity [60]. Nonetheless, intermyocellular and perimuscular adipose tissues (IMAT and PMAT) might mediate paracrine effects on skeletal muscle [61], and nutrient overload resulting in extracellular (i.e., enhanced local pro-inflammatory cytokine production [62], enhanced angiotensin II activity [63], decreased adiponectin autocrine/paracrine effects [64]) and intracellular events (mitochondrial oxidative damage [65], free fatty acid accumulation [66]) may altogether contribute to impaired insulin sensitivity in adipose tissue, which in turn might further aggravate lipid spillover through defective insulin-mediated inhibition of lipolysis [67].

**Box 1:**
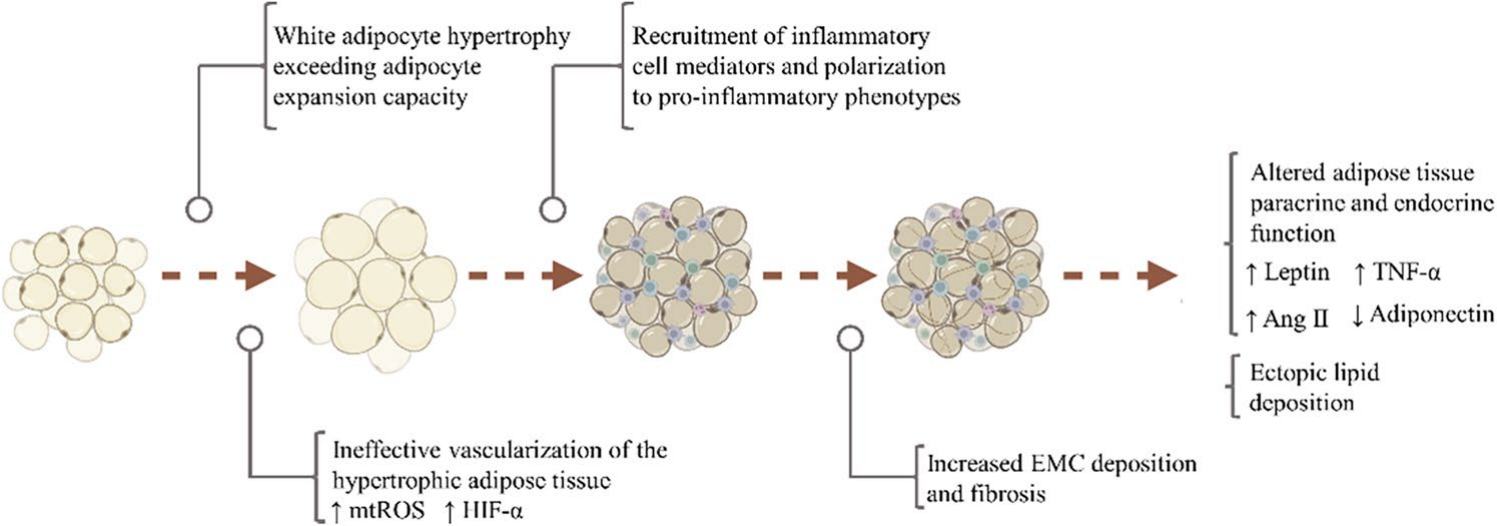
Contributory role of adipocyte hypertrophy to metabolic dysregulation

Overnutrition coupled with physical inactivity is causative of white adipocyte hypertrophy through triglyceride accumulation [68, 69], which may be considered an early event in the metabolic dysregulation associated to obesity. Hypertrophic adipocytes are dysfunctional, presenting ultrastructural abnormalities and limited hyperplastic expansion capacity [70, 71], which compromises their lipid storage function and may result in lipid overflow to non-adipose tissues [72]. Exacerbated adipose growth also challenges the vascularization of the growing tissue resulting in hypoxia [73], which triggers the production of mitochondria-derived reactive oxygen species (mtROS) [74], and mediates the overexpression of hypoxia-inducible factors (HIF) [75]. Accumulation of macrophages and lymphocytes along with polarization to pro-inflammatory phenotypes, fatty acid overload, and persistent inflammation may underlie EMC deposition and ensuing fibrosis, thus further compromising both storage and endocrine functions [76]. Changes in production of cytokines/adipokines derived from adipocytes and immune cells (i.e., leptin, adiponectin, tumor necrosis factor α (TNF-α), angiotensin II (Ang II), etc.) contribute to metabolic dysregulation and persistent low-grade inflammation in obese patients, which has been associated with compromised insulin signaling and protein turnover in skeletal muscle [77].

T2DM is characterized by insulin resistance in combination with β-cell dysfunction. While hyperinsulinemia has been typically regarded as a compensatory mechanism triggered in response to IR, enhanced β-cell proliferation and increased insulin secretion, which characterize diabetogenic processes, have been reported to precede IR in obesity and under obesogenic conditions [78, 79]. Although the interplay between β-cell dysfunction and IR is intricate, overt T2DM associates with reduced β-cell mass due to enhanced protein degradation [80], leading to inability to compensate sustained insulin resistance, and hyperglycemia [81]. Excess glucose availability and increased insulin production promote hepatic *de novo* lipogenesis, which aggravates hepatic lipid accumulation, and consequently enhances gluconeogenesis, therefore contributing to systemic hyperglycemia and β-cell dysfunction [82]. Long-term elevation in glucose levels is considered the main culprit of β-cell demise and dysfunction due to impaired calcium handling and ROS/inflammation-mediated mechanisms, which combined with lipid accumulation and impaired lipid metabolism is termed “glucolipotoxicity” [83]. Particularly, β-cells are highly susceptible to oxidative damage due to their limited antioxidant capacity [84], being ROS generation and mitochondrial damage proposed as major mechanisms driving glucolipotoxicity-induced β-cell dysfunction [85].

Hyperglycemia represents a key mediator in the transition from obesity to T2DM through IR and β-cell dysfunction, and is positioned as a central factor in the pathogenesis of skeletal muscle atrophy in T2DM patients [86]. Non-controlled hyperglycemia promotes irreversible formation of advanced glycation end products (AGEs) through non-enzymatic processes, whose accumulation in musculoskeletal tissues compromises their biomechanical properties and promotes the interaction with cell membrane receptors (e.g., receptor for advance glycation end products (RAGE)), thus triggering intramyocellular events involved in processes of atrophy (e.g., inflammation and ROS generation, etc.) [87]. Not only hyperglycemia but also impaired nitric oxide (NO) production in skeletal muscle arterioles through endothelial nitric oxidate synthetase (eNOS)-dependent pathways is a critical consequence of peripheral IR [88]. Further, elevations in endothelium-derived entohelin-1 (ET-1) correlate with IR in humans and may directly impact the skeletal muscle through inhibited insulin-mediated glucose uptake [89], with recent evidence pointing to a potential direct impact of ET-1 on skeletal muscle atrophy through pro-inflammatory signaling pathways [90]. Decrements in NO levels combined with enhanced ET-1 activity operate to decrease blood flow to skeletal muscle tissue, and, consequently, compromise nutrient delivery, including amino acids needed for skeletal muscle protein turnover [91].

**Fig. 1:**
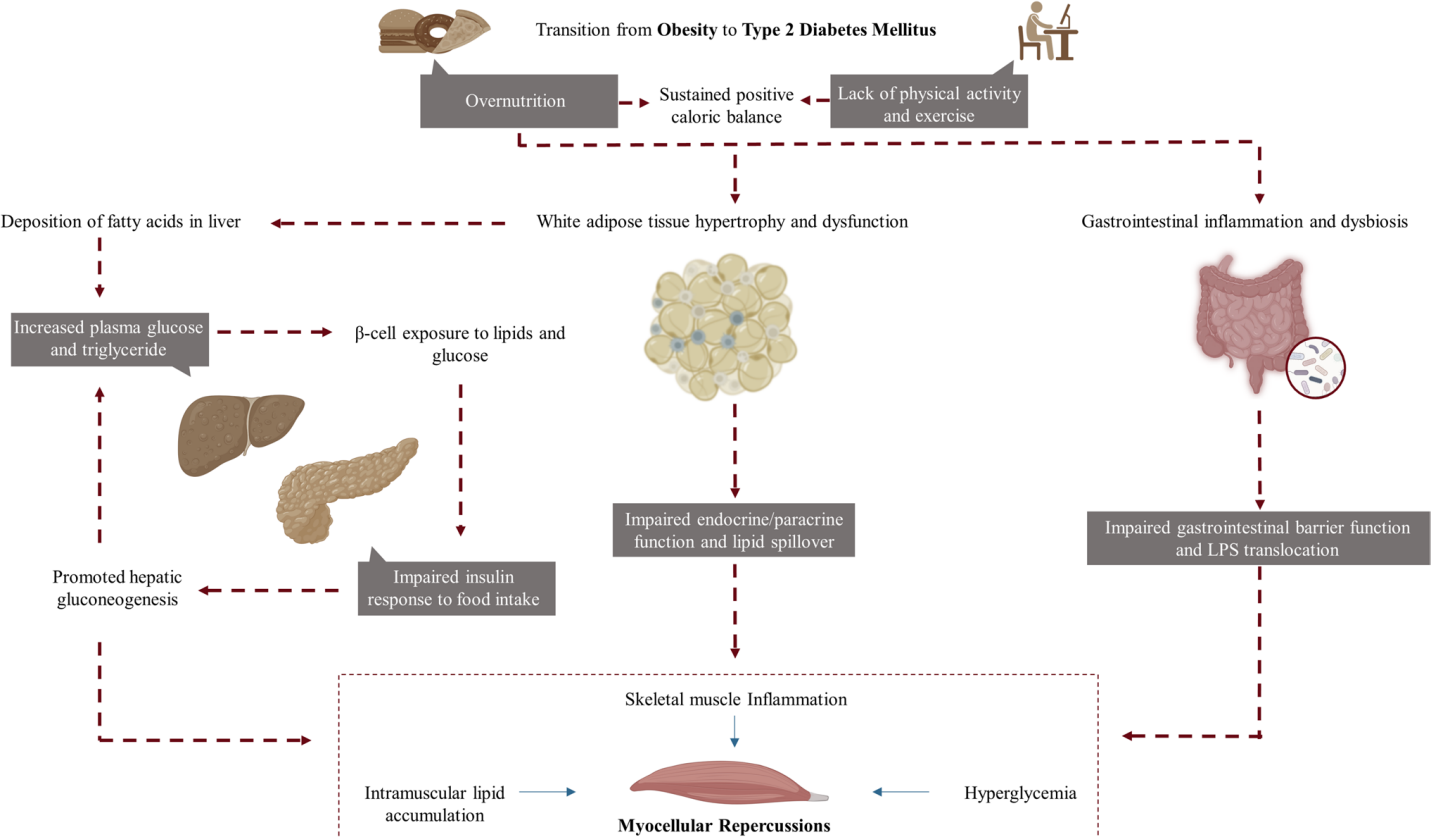
Main metabolic signatures of obesity and type 2 diabetes mellitus involved in musculoskeletal health

Lastly, shifts in the composition of the gut microbiota observed with obesity and T2DM are considered to contribute to low-grade inflammation [92]. Whereas core sets of microorganisms defining “healthy” or “unhealthy” gut microbiota profiles are far from delineated, enhanced intestinal permeability is consistently documented both in obesity and T2DM [93, 94], which is linked to lipopolysaccharide (LPS) endotoxemia [95]. LPS is a potent proinflammatory mediator contained in gram-negative bacteria outer membrane, and capable of inducing pro-catabolic state in skeletal muscle [96]. In addition, gut dysbiosis is associated with differences in metabolomic profiles of gut bacterial byproducts with documented systemic effects in insulin signaling and muscle metabolism [97]. Some of these metabolites, most notably short-chain fatty acids (SCFAs), are also implicated in the remodeling and maintenance of the gut barrier [98]. However, the intricate interplay between gut bacteria metabolites and the skeletal muscle – oftentimes referred to as “the gut-muscle axis” – in the context of metabolic disorders, such as obesity and T2DM, remains an emerging topic of research, and further studies are needed to delineate mechanisms at play. A schematic representation of the main metabolic signatures of obesity and T2DM involved in musculoskeletal health is presented in Fig. 1.

## Myocellular repercussions of obesity and T2DM

Skeletal muscle atrophy and dysfunction are typically discussed in terms of decrements in mass and quality. While skeletal muscle mass refers collectively to all body’s skeletal muscle tissue, muscle quality is a multi-dimensional aspect of skeletal muscle, and is typically measured as strength relative to muscle mass [99]. Different dimensions have been proposed to integrate muscle quality including skeletal muscle composition, architecture, and ultrastructure [99]. However, metabolic, thermoregulatory, autocrine, paracrine, and endocrine domains may be also considered within this framework [100]; therefore, skeletal muscle quality should be evaluated from a broader viewpoint extending beyond relative strength.

It is important to mention that most mechanisms involved in changes of muscle properties associated with obesity and T2DM are centralized through insulin signaling, a key player in the transition from obesity to T2DM, due to its profound impact on skeletal muscle anabolism/catabolism, and metabolism [51] (Box 2). The question herein is how impaired insulin signaling translates to compromised skeletal muscle mass. Current consensus supports that insulin has a permissive effect on skeletal muscle anabolism when amino acids are sufficiently available [101]. In fact, IR is proposed to contribute to an age-related decline in muscle protein synthesis (MPS) in response to anabolic stimuli, particularly amino acid feeding, a phenomenon termed “age-related anabolic resistance” [102, 103]. Studies using stable isotope tracer methodologies have indicated that insulin anabolic effects on skeletal muscle are largely mediated through endothelial-dependent vasodilation, involving modulation of endothelial-derived signaling molecules namely NO and ET-1 [91]. Consequently, insulin stimulates nutrient delivery to the skeletal muscle, including amino acids that promote MPS through mTORC1 signaling [91]. Perhaps more important is the role of insulin in attenuating skeletal muscle catabolism. As mentioned, insulin controls the expression of atrogenes (i.e., UPS E3 ligases, autophagy) involved in muscle protein breakdown (MPB), mainly through Akt signaling. Together with the contributory role of Akt-independent activation of MPB through MAPK pro-inflammatory signaling, IR may induce skeletal muscle atrophy in insulin resistant obese individuals, and T2DM patients through unbalanced muscle protein turnover denoted by exacerbated MPB, and potentially impaired MPS response to nutritional anabolic stimuli. Lastly, it is worth noting that skeletal muscle atrophy may result in the selective loss of proteins with potential effects on muscle functionality, which has been scarcely explored. For example, aquaporins (e.g., aquaporin 4 (AQP4)), have been implicated not only in water transport but also in metabolism and fatigue resistance [104], and are markedly targeted for degradation under muscle atrophy [105].

**Box 2:**
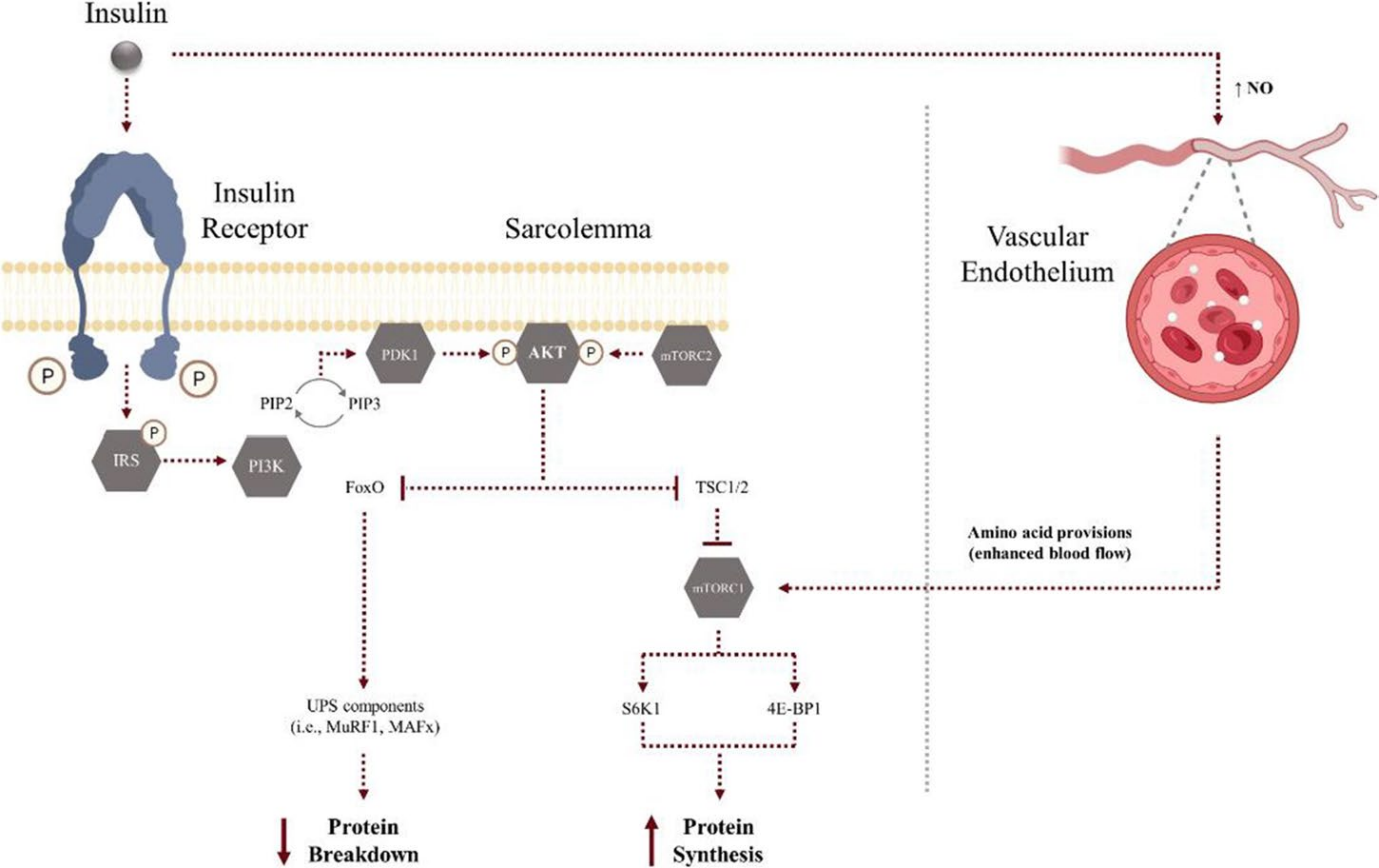
Insulin signaling in skeletal muscle anabolism and catabolism

Insulin exerts a wide range of effects not only on skeletal muscle metabolism but also anabolism and catabolism through modulation of the insulin receptor-Akt signaling pathway. Briefly, the binding of insulin to its surface receptor triggers the recruitment and phosphorylation of insulin receptor substrates (IRS) mainly IRS1 and IRS2. Phosphorylation of IRS1 proteins creates binding sites for the regulatory subunit of phosphatidylinositol 3-kinase (PI3K). The second messenger phosphatidylinositol-3,4,5-triphosphate (PIP3) docks pleckstrin homology (PH) domains of Akt and phosphoinositide-dependent kinase 1 (PDK1), thus facilitating their activation and migration to the cell membrane [106]. Following membrane relocation, Akt is partially activated by PDK1 and, subsequently, activates the mechanistic target of rapamycin (mTOR) complex 2 (mTORC2) to facilitate a further Akt phosphorylation, leading to full activation [107]. Akt plays a central role in modulating distant insulin signaling effects at different levels, being the most representative process insulin-stimulated glucose uptake. Specifically, Akt is both necessary and sufficient to facilitate insulin-mediated translocation of the skeletal muscle glucose receptor GLUT4 and fusion with the plasma membrane, thus enabling muscle glucose uptake [108]. Other major metabolic pathways include those involved in fatty acid uptake and glycogen synthesis (reviewed in ref. [109]). In the context of skeletal muscle atrophy, the role of Akt in regulating skeletal muscle protein turnover is critical. Phosphorylation of tuberous sclerosis complex 2 (TSC2) by Akt facilitates mTORC1 activation at the lysosome. Activation of mTORC1, in turn, leads to phosphorylation of different substrates and downstream targets (e.g., translational initiation promotor ribosomal protein s6 kinase-1 (S6K1) and eukaryotic initiation factor (4E-BPI)) known to positively regulate translational machinery to trigger protein synthesis, cell growth, and proliferation [110]. Not only is mTORC1 considered a master regulator of protein synthesis but is also known to suppress cell catabolism through inhibition of multiple autophagy-related proteins (reviewed in ref. [110]). However, disrupted Akt signaling appears to be more relevant than mTORC1-mediated control of autophagy in settings of skeletal muscle atrophy [111]. Akt is demonstrated to negatively phosphorylate forkhead box class O (FoxO) transcription elements, particularly the skeletal muscle predominant isoforms FoxO1 and FoxO3, which facilitates their relocation to the cytoplasm and represses the expression of targeted downstream genes involved in the activation of ubiquitin-proteasome system (UPS) E3 ubiquitin ligases, mainly muscle RING finger 1 (MuRF1) and Atrogin-1/muscle atrophy F-box (MAFbx) [112], as well as different genes involved in autophagy/lysosomal pathways. The UPS involves the coordinated action of three enzymes (E1, E2, and E3) that operate to target short-lived/damaged proteins through ubiquitination in order to facilitate their degradation by the 26S proteasome complex, being MAFbx and MuRF1, two critical skeletal muscle-specific E3 ubiquitin ligases consistently described to be upregulated under atrophic conditions [113]. While FoxOs are thought to be primarily modulated by Akt, positive phosphorylation of FoxOs by mitogen-activated protein kinases (MAPKs) in Akt-independent manner has been described, which might also contribute to enhanced protein degradation in response to cellular stress (discussed in later sections) [114, 115].

### Intramyocellular lipid accumulation

As mentioned before, ectopic lipid accumulation, particularly to skeletal muscles, may be a key contributor to IR under chronic overnutrition. SCFAs and most medium-chain fatty acids (MCFAs) readily cross cellular and mitochondrial membranes. In contrast, long-chain fatty acids (LCFAs) require fatty-acid binding proteins for cellular uptake, intracellular transport, and metabolism [116]. Upregulation of the skeletal muscle transmembrane fatty acid translocase FAT/CD36 has been reported in obese and T2DM individuals [117, 118]. Other putative sarcolemmal fatty acid transporters include the plasma membrane-associated fatty acid binding protein (FABPpm) and fatty acid transporters 1 and 4 (FATP1,4) [119]. Sarcolemmal translocation of all these transport proteins has been shown to be modulated by insulin [120], and several research lines suggest an impact of diabetogenic conditions on their translocation and expression in skeletal muscle [121, 122], albeit the individual contribution of each transporter remains ill-defined. Fatty acids are subsequently trapped within the muscle cell through conversion to fatty acyl-CoA esters mediated by acyl-CoA synthetases as well as through binding to the cytosolic binding protein FABPc [123], and mobilize to metabolic sites. Early studies observed a reduction in enzymatic oxidative pathways in mitochondria of T2DM patients [124] while more recent observations suggest that decreased mitochondrial content and impaired dynamics may underlie mitochondrial dysfunction and compromised fatty acid oxidation in obesity and diabetes (discussed in later sections) [125]. Promoted fatty acid cellular uptake coupled with mitochondrial dysfunction (impaired disposal) contribute to IMCL accumulation in skeletal muscle.

Among IMCLs, intramuscular triacylglycerides (IMTGs) were originally thought to the main culprit of IR resulting from lipid oversupply. However, accumulation of IMTGs may be considered to play a protective role aimed to prevent increases in different lipotoxic metabolites, most notably diacylglycerols (DAGs) and ceramides. DAGs are lipidic compounds that originate from multiple sources including breakdown and *de novo* synthesis of triglycerides [126]. Particularly, sn-1,2-DAGs stereoisomers have been extensively implicated in the onset of IR through recruitment/activation of atypical protein kinase-c (PKC) isoforms, particularly PKCε and PKCθ [127], yet associations of intramuscular DAGs and IR are not always consistent and might depend on length and saturation of DAG fatty acid chains, and divergent subcellular locations [128]. In this sense, recent research suggested that sn-1,2-DAGs contained in the skeletal muscle cell membrane might mediate IR [129], which consequently phosphorylate the insulin receptor substrate-1 (IRS-1) thus blocking downstream Akt activation, a key node in the insulin signaling cascade, and attenuating insulin signaling [130]. In support of these findings, overexpression of diacylglycerol kinase δ (DKG-δ), an isoform highly expressed in skeletal muscle of the enzyme that catalyzes the conversion of DAG to phosphatidic acid, has been shown to protect against high-fat diet-induced glucose intolerance in skeletal muscle [131, 132].

Another family of compounds argued to contribute to IR in skeletal muscle consists of ceramides, particularly C18 species [133]. Ceramides are synthetized via different pathways, including *de novo* synthesis in endoplasmic reticulum (ER) from serine and an acyl-CoA, preferentially palmitate, though the rate-limiting step enzyme serine palmitoyltransferase (SPT), to later undergo reduction, and an acetylation step responsible for their fatty acid chain composition [134]. Further reactions lead to different members of the family [134]. In settings of saturated FA oversupply, ceramide transporter (CERT) activity is attenuated, which compromises ceramide transport from ER to Golgi apparatus and promotes its accumulation [135]. Notably, ceramides are demonstrated to inhibit Akt in skeletal muscle by activating PKCζ and protein phosphatase 2A (PP2A) resulting in impaired insulin signaling [136, 137]. Of note, long-term exposure to ceramides may involve inhibition of IRS-1 though activation of the double-stranded RNA-activated protein kinase (PRK)/ cJun-N-terminal kinase (JNK) proinflammatory pathway [138]. Overall, compelling evidence supports a role of certain intramuscular toxic lipid species in the disruption of skeletal muscle insulin signaling in obesity, in turn compromising skeletal muscle mass and metabolism.

### Skeletal muscle inflammation

Besides lipotoxicity induced by intramyocellular accumulation of above-mentioned lipid species, a solid body of evidence suggests that excess fatty acid supply might also mediate IR through pro-inflammatory mechanisms. Particularly, expanded IMAT and PMAT are considered major sources of pro-inflammatory factors in skeletal muscle though paracrine modulation [61, 139]. In line with visceral adipose tissue, polarized M1-like macrophages and T-cells, particularly Th1 cells, accumulate preferentially to IMAT and PMAT and promote the production of proinflammatory cytokines resulting in myocyte inflammation associated with IR [61]. On the other hand, local production of cytokines in the skeletal muscle (myokines) is closely linked to the acute effect of exercise, and myokines are typically attributed many of the beneficial effects of exercise on metabolism, including glucose homeostasis [140]. While a potential contributory role of persistent autocrine inflammation in skeletal muscle may not be discarded [141], an immune cell origin of pro-inflammatory mediators from adjacent adipose tissue appears to play a protagonist role in skeletal muscle inflammation.

Several pro-inflammatory factors have been proposed to compromise insulin signaling in obese skeletal muscle. Among them, TNF-α is probably the most extensively studied cytokine demonstrated to induce IR through activation of the TNF receptor 1 (TNFR1) [58, 142]. Thereinafter, several intracellular steps involving the formation of protein complexes, interaction with scaffolding kinases, and generation of ubiquitin chains take place to facilitate the interaction with the downstream MAPKs JNK, p38, and extracellular signal-regulated kinase (ERK) (a known IRS-1 kinase [143]) as well as the activation of the inhibitor of nuclear factor κB (IKK)/NF-κB pathway [144] – IKK promotes the degradation of the NF-κB inhibitor IκB in TNF-α-dependent manner [145] – thereby mediating disruption of IRS-1 in skeletal muscle [146, 147]. Other pro-inflammatory factors interact with MAPKs/NF-κB and might mediate IR in skeletal muscle. For instance, chemokines such as the leukotriene B4 (LTB4), have been shown to promote IR in myocytes through JNK-dependent mechanisms [148]. IL-1β is also demonstrated to interact with MAPK and NF-κB [149], and has been documented to mediate IR in different tissues [150–152]. Not only immune cell-derived cytokines but also circulating LPS can mediate interaction with MAPK and NF-κB through recognition/agonism of TLR4. Besides abovementioned effects of toxic lipid species, such as DAG and ceramides, on insulin signaling, saturated FAs (e.g., lauric acid and palmitic acid) are demonstrated to activate innate immune responses through interaction with TLR4 and TLR2 [153–155]. Interaction with TLRs can, in turn, contribute to IR through above-cited pro-inflammatory pathways.

IL-1β has gained great attention in recent years due to the potential role of the NLRP3 inflammasome, a protein complex responsible for the activation of pro-inflammatory IL-1β, in mediating insulin resistance in skeletal muscle. NLRP3 activation induced by diet has been consistently shown to interfere with insulin signaling in different tissues [156], including skeletal muscle [157, 158], and mechanisms involved in NLRP3-mediated IR remain an interesting field of research. Notably, NLRP3 is triggered through activation of TLR4, and stimulation of TLR4 by physiological levels of bacterial LPS in myocytes has been shown to promote mitochondrial dysfunction and mtROS production [159], which denotes the complex interaction between inflammatory and oxidative components, and supports a potential role of other circulating pro-inflammatory mediators in skeletal muscle IR.

Interferon γ (IFN- γ) produced by pro-inflammatory Th1 cells infiltrating to IMAT and PMAT is another potential contributor to skeletal muscle IR [61, 160]. Upon binding to the interferon receptor (IFN-γR1/2), Janus kinases JAK1 and JAK2 are stimulated, which induce phosphorylation and activation of signal transducer and activator of transcription (STAT) elements, primarily STAT1. Subsequently, STAT1 relocates to the nucleus and mediates transcription of genes involved in immune and inflammatory responses such as suppressor of cytokine signaling (SOCS) molecules [161]. Further, IL-6, a cytokine generally considered pro-inflammatory under obesogenic conditions, was demonstrated to activate STAT3 in myocytes and promote ubiquitination and degradation of IRS1 resulting in IR [162]. In alignment, chronic activation of the IL-6-JAK-STAT3 pathway in skeletal muscle has been shown to promote mtROS production, which in turn further enhanced STAT3 signaling and resulted in mitochondrial dysfunction and cellular oxidative stress [163].

Lastly, as noted above, inflammation related pathways may contribute to skeletal muscle atrophy in obesity and T2DM through insulin-independent mechanisms, involving activation of MAFbx and MuRF1 E3 ubiquitin ligases. NF-κB transcription factors p50 and Blc-3 were shown to be required for disuse-induced atrophy, and associated with enhanced expression of MuRF1 and MAFbx [164]. Further, increased expression of MuRF1, but not MAFbx, along with signs of muscle atrophy have been observed with induction of NF-κB in rodent models [165]. Induction of MAFbx in skeletal muscle by TNF-α exposure was shown to be mediated by p38 MAPK, but not JNK and ERK1/2 [166]. In same fashion, IL-6-mediated activation of STAT3 was shown sufficient to induce skeletal muscle atrophy *in vitro* and *in vivo* together with elevated expression of SOCS3 and MAFbx [167]. Moreover, STAT3 was observed to enhance expression of myostatin, a well-documented negative regulator of muscle growth, through activation of the transcription factor C/EBPδ in skeletal muscle [168]. Overall, skeletal muscle inflammation not only associates with IR but also with a promoted activation of protein degradation systems, which might contribute to unbalanced protein turnover and atrophy.

### Mitochondrial dysfunction and oxidative stress

Decrements in skeletal muscle mitochondrial content have been observed in individuals with obesity, and in T2DM patients [169]. Lower mitochondrial content in these populations has been shown to associate with a reduction in the expression of genes involved in mitochondrial biogenesis, particularly the peroxisome proliferator-activated receptor-gamma coactivator (PGC-1α) [170, 171], which is markedly expressed in oxidative (I and IIa) compared to glycolytic fibers (IIx) [172]. Saturated fatty acid feeding is documented to induce PGC-1α expression in lean subjects, but sustained overfeeding might exhaust PGC-1α signaling in obese individuals [173]. In alignment, lipid infusion has been reported to decrease skeletal muscle PGC-1α expression [174]. Among different upstream regulators, PGC-1α is activated by AMP-activated protein kinase (AMPK), a central fuel-sensing enzyme that is activated in cellular situations of elevated AMP:ATP ratio, which results in the transcription of different nuclear factors known to promote mitochondrial biogenesis processes [175]. Overnutrition shuts down AMPK signaling leading to impaired PGC-1α, which may partially underlie decrements in mitochondrial content.

Balance between mitochondrial fusion and fission processes is critical to ensure adequate mitochondrial quality control. However, an increasing number of studies have reported fragmented mitochondrial networks and disrupted mitochondrial rhythm (i.e., circadian changes in mitochondrial dynamics and oxidative capacity [176, 177]) in skeletal muscle tissue in obesity and T2DM [178–180]. Promoted skeletal muscle expression of fission-related proteins (fission protein 1 (Fis1) and dynamin-related protein-1 (Drp-1)) occurs with palmitate treatment and high-fat diet consumption [181, 182], and associates with decreased insulin-mediated glucose uptake [182]. Overexpression of Drp-1, specifically, has been consistently shown to associate with mitochondrial fragmentation in obesity and T2DM [183]. In alignment, loss of Drp-1 in myocytes from obese insulin-resistant subjects was demonstrated to enhance insulin action and improve mitochondrial network morphology [184]. Further, lipid infusion has been documented to promote activation of Drp-1 and accumulation of PTEN-induced putative kinase-1 (PINK1), a protein involved in mitochondrial removal (mitophagy), in skeletal muscle of sedentary healthy subjects, which was also linked to increased mitochondrial fragmentation and impaired peripheral glucose disposal [185]. It is worth noting that ceramide accumulation has also been reported to promote mitochondrial fission via increased expression of Drp-1 [186, 187]. Mitochondrial accumulation of ceramides might be particularly detrimental for mitochondrial function since ceramides are reported to induce depletion of several electron transport chain complexes, and may be incorporated to mitochondrial membranes thus facilitating proton leakage [188, 189]. Based on these observations and supported by evidence from other knock-out/knock-down experiments, the pharmacological inhibition of Drp-1 has recently garnered attention as a therapeutic tool for obesity and T2DM [190].

Decreased expression of mitochondrial fusion elements might contribute to pro-fission phenotypes in skeletal muscles. Mitofusin 1 and 2 (MFN1 and MFN2) are transmembrane proteins responsible for the tethering of mitochondrial outer layers, thus playing a crucial role in mitochondrial fusion [191]. Particularly, MFN2 is highly expressed in skeletal muscle, and its expression has been shown to decrease in obese rodents and human subjects with obesity, and T2DM [181, 192, 193]. Interestingly, some authors have suggested that promotion of Drp-1 might precede altered expression of fusion-related proteins [194]. Nonetheless, some evidence suggests that compensatory mechanisms involving secretion of myokines (e.g., fibroblast growth factor 21 (FGF-21) with insulin-sensitizing properties might occur with Drp-1 deficiency due to endoplasmic reticulum (ER) stress, which highlights the complex interplay between mitochondrial dynamics and cell homeostasis [195, 196]. The removal of fragmented mitochondrial networks resulting from pro-fission phenotypes is key to facilitate quality control, and limited research suggests that enhanced mitophagy mechanisms might compensate promoted mitochondrial fragmentation in settings of cellular fuel oversupply [197]. However, science on the effects of obesity and diabetes on skeletal muscle mitophagy is in its infancy and further research is needed [125]. Overall, smaller and less abundant mitochondria in skeletal muscle of obese and T2DM individuals appear to be at least partially driven by compromised biogenesis, increased fragmentation of mitochondrial networks and, potentially, impairments in defective mitochondria removal mechanisms.

Sustained oxidative stress is considered as a key consequence of mitochondrial dysfunction. Nutrient overload in settings of low ATP demands puts an important strain on the electron transport chain (ETC) and promotes chronic production of mtROS such as peroxide (H_2_O_2_). ROS production may, in turn, potentially exceed the capacity of antioxidant mechanisms, and lead to increased generation of partially oxidized lipid intermediates [198]. Mitochondrial fragmentation has been documented to increase mtROS [199], and enhanced H_2_O_2_ may also may reciprocally contribute to mitochondrial fragmentation [200]. Importantly, mtROS production is a common factor in different IR models, and findings from different studies have supported a causative role of mtROS, particularly H_2_O_2_ [201, 202], in triggering IR [65, 203]. Further, overexpression of antioxidant enzymes has been shown to exert a protective effect against diet-induced IR mediated by mtROS in skeletal muscle [202, 204, 205].

**Fig. 2:**
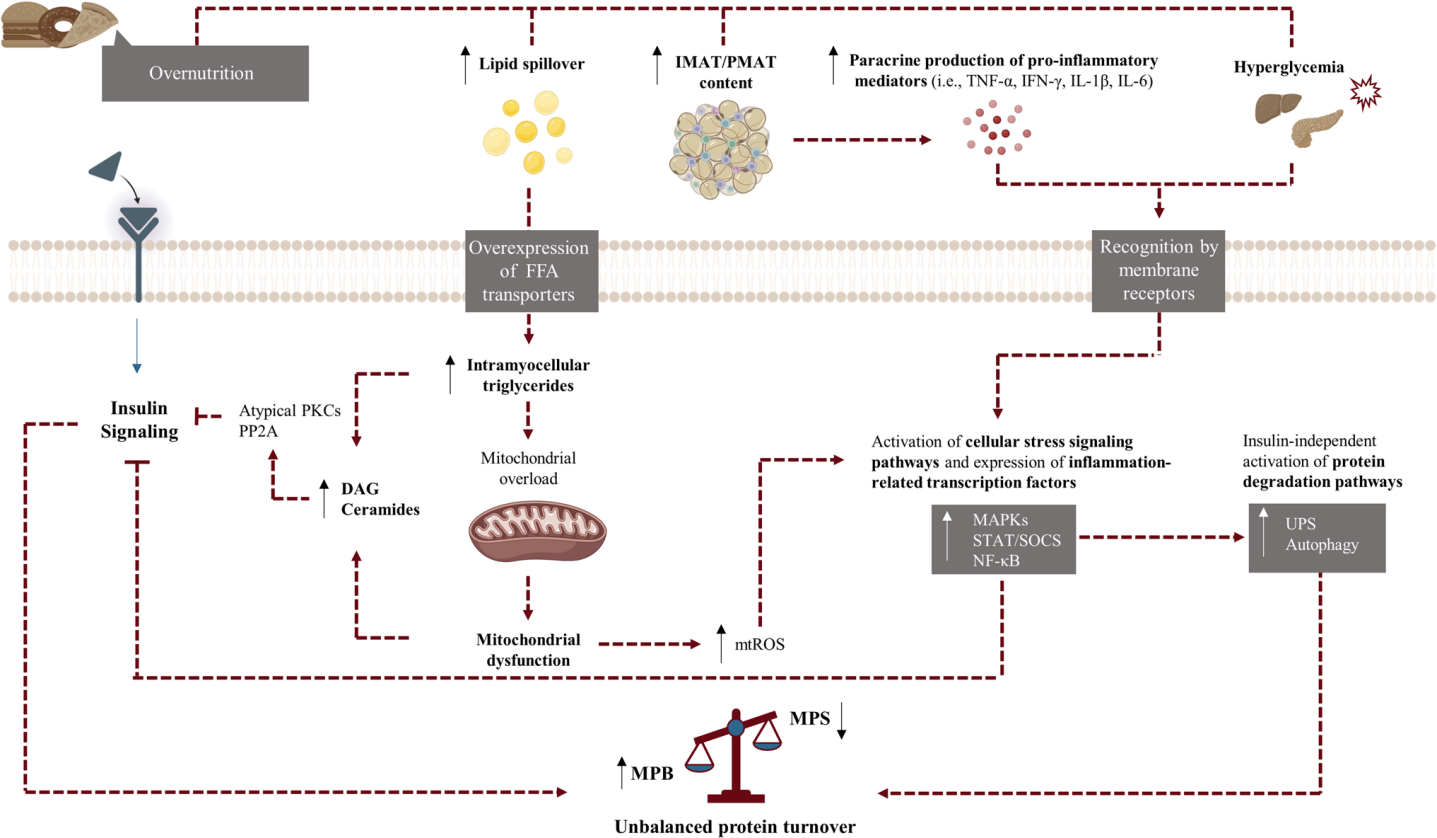
Interplay between cellular stress signaling pathways, insulin resistance and skeletal muscle protein turnover. DAG, diacylglycerol; FFA, free fatty acids; IFN- γ, interferon γ; IL, interleukin; IMAT/PMAT, intermuscular/perimuscular adipose tissue; MAPK, mitogen activated protein kinases; MPB, muscle protein breakdown; MPS, muscle protein synthesis; mtROS, mitochondrial reactive oxygen species; NF-κB, nuclear factor κB; PKC, protein kinase C; PP2A, protein phosphatase 2A; SOCS, suppressor of cytokine signaling; STAT, signal transducer and activator of transcription; TNF-α, tumor necrosis factor α; UPS, ubiquitin proteasome system

Mechanisms underlying mtROS-mediated deleterious effects on skeletal muscle insulin actions are complex and involve the activation of several cellular metabolic stress sensors, most notably the extensively studied MAPK pro-inflammatory pathways. Probably, the most well-documented MPAK involved in ROS-mediated effects on insulin signaling is JNK. In settings of sustained oxidative stress, JNK1/2 isoforms are chronically activated, and activation of JNK1/2 leads to phosphorylation of IRS-1 and IRS-2, thus negatively regulating insulin actions [206, 207]. Mitochondrial ROS also favor the activation of p38 MAPK, and, although controversial, some evidence supports a potential role of p38 MAPK on mtROS-mediated impairment of insulin-induced glucose uptake [208, 209]. Besides, other redox-sensitive components with potential implications in insulin signaling include NF-κB, a pleiotropic transcription factor that upregulates cytokine and chemokine production and may be involved in several processes of muscle atrophy, as well as activation of the NLRP3 inflammasome [157]. It should be noted that ROS produced from non-mitochondrial sources might also participate from activation of cited proinflammatory cascades. Particularly, an increasing body of evidence supports a role of angiotensin II in mediating phosphorylation of IRS-1 in skeletal muscle through a NADP-ROS-NF-κB -dependent pathway [210, 211]. Sustained oxidative stress associates with oxidative damage, and results in generation of oxidated molecules (e.g., lipid peroxides) and by-products. Particularly, the lipid peroxidation by-product 4-hydroxy-2-nonenal (4-HNE) has been shown to impair insulin sensitivity in skeletal muscle cells, which may be mediated by formation of adducts on IRS-1 and Akt, or acting as second messenger in MAPK-related pathways [212, 213]. Altogether, several lines of research support a contributory role of mitochondrial dysfunction in the onset of skeletal muscle IR in obesity and T2DM progression through the sustained generation of mitochondrial ROS. Further, a potential loss of mitochondrial plasticity in obesity and diabetes might compromise skeletal muscle metabolic flexibility in these individuals [214], with consequent implications in muscle functionality. A brief summary of how cellular stress signaling pathways and insulin resistance operate to compromise balance in skeletal muscle protein turnover in the context of chronic overnutrition is depicted in Fig. 2.

### Skeletal muscle fiber shift

The study of skeletal muscle fiber phenotypes is complex due to different challenges including the existence of intermediate/hybrid human fiber types featuring different proportions of myosin heavy chain (MHC) isoforms, differences between human and rodent skeletal muscle fibers used in experimental models, differences in factors involved in developmental versus adult transition/reprogramming processes, and limitations of the methodological approaches used to characterize them [215]. Nevertheless, slow-twitch type I fibers have been documented to possess increased GLUT-4 protein content, promoted expression of various components of the insulin signaling cascade, and higher insulin sensitivity compared to fast-twitch fibers [29, 216–218]. They also have higher mitochondrial content, rely on mitochondrial oxidation as their main energy source [219], making them better equipped to handle excess fat supply, and are more resistant to atrophic conditions [219]. Therefore, the slow-to-fast shift in muscle fibers seen in obese [220] and diabetic individuals [27–30] is considered to negatively contribute to the progression of these metabolic conditions, and might be linked to the onset of IR in obese patients [217].

Signaling components involved in skeletal muscle fiber transition have been subject to extensive research. Overall, compelling evidence suggests that compromised content of skeletal muscle oxidative fibers may be partially attributed to impaired AMPK-PGC-1α signaling. As commented, overnutrition in absence of exercise leads to inhibition of AMPK-PGC-1α signaling. PGC-1α has been reported to directly activate several myocyte enhancer factor-2 (Mef2) proteins (particularly Mef2C and Mef2D), which are transcription factors known to promote the transition to oxidative fibers [221, 222]. Further, PGC-1α activates peroxisome proliferator-activated receptor β (PPARβ) (formerly PPARδ), which has been demonstrated to promote oxidative fiber shift [223, 224]. Adiponectin levels are decreased in obesity and T2DM, and activation of the skeletal muscle adiponectin receptor adipoR1 is known to upregulate PGC-1α through AMPK [225]. In the same vein, palmitate has been shown to stimulate folliculin interacting protein 1 (FNIP1) expression in skeletal muscle which is known to bind and repress AMPK activity, thus inhibiting fiber oxidative phenotypes [226]. In light of the evidence, targeting upstream AMPK regulators (i.e., Sirtuin 1 (SIRT1), adipoR1) through nutritional interventions aimed to reverse obesity- and T2DM-mediated conversion to type-II muscle fibers is increasingly pursued [227, 228].

Calcium signaling is critical for skeletal muscle fiber transition, particularly in response to exercise. Briefly, sustained muscle contraction elevates Ca^+2^ levels in muscle fibers, which are sensed by components of two signaling pathways involved in Mef2 activation, namely calmodulin-calcineurin complex and calcium/calmodulin-dependent protein kinase (CaMK). Binding of Ca^+2^ to the calmodulin-calcineurin complex activates the phosphorylation and translocation to the nucleus of nuclear factor of activated T cells (NFAT), particularly NFATc, which promotes Mef2 transcription [229]. Further, mentioned Ca^+2^-dependent mechanism are documented to interact with PGC-1α signaling through coordinated changes in PGC-1α and PPARβ expression. Importantly, some evidence suggests that skeletal muscle Ca^+2^ handling might be impaired in obesity and T2DM through mechanisms involving ROS-mediated ryanodine receptor (RyR) inhibition, and mitochondrial dysfunction/impaired mitochondrial Ca^+2^ regulation [230], which might also partially underlie compromised muscle contraction capacity in these populations. It is also worth mention that type-I oxidative fiber are less-fatigable, but generate less force than type-II glycolytic fibers, which should theoretically imply higher skeletal muscle power output in obese individuals without signs of skeletal muscle mass loss [231]. Nonetheless, impaired calcium handling coupled with the potential deleterious effects of IMCL species on myosin binding protein-C (cMyBP-C) contractile properties have been proposed to partially explain impaired contractile function in obese subjects [232].

### Impact of hyperglycemia on skeletal muscle

When increased insulin secretion in obese individuals fails to regulate glucose levels, hyperglycemia occurs, which is closely linked to aggravated IR and compromised β-cell function, thus being considered a major factor in the transition to T2DM. Sustained hyperglycemia in T2DM contributes to the formation of endogenous advanced glycation end products (AGEs), which consist of a diverse group of compounds generated through non-enzymatical reactions between carbonyl groups of reducing sugars and free amino acid groups of different body molecules [233]. Some early glucose-derived AGEs include well-described markers of glucose homeostasis such as glycated hemoglobin (Hb1AC) and fructosamine [233]. Further rearrangements facilitate the conversion of early AGEs into irreversible molecules, which are reported to accumulate in T2DM patients and associate with T2DM complications [234]. While AGEs encompass a highly heterogenous set of compounds – generally classified based on their chemical structure and ability to emit fluorescence – Nε-carboxymethyllysine (CML) (the dominant circulating AGE), Nε-carboxyethyllysine (CEL), and pentosidine are among the most frequently studied in the context of diabetes [235].

AGEs may play important roles in processes of skeletal muscle atrophy and dysfunction. AGE-induced modifications of skeletal muscle proteins, such as myosin and actin, might impair their contractile properties leading to loss of muscle strength [236]. Long-lived EMC proteins, particularly collagen, might be especially susceptible to AGE glycation and cross-linking, which in turn may result in structural disarrangements, disrupted binding affinity, and stiffness [237]. However, not only structural damage but also binding to cell receptors is reported to mediate AGE-induced impairments in skeletal muscle mass and quality. The receptor for advanced glycation end products (RAGE) is a multiligand receptor capable of recognizing several AGEs, s100 proteins, and high mobility group B1 (HMGB1) as well as pathogen associated molecular patterns, such as LPS [238]. Previously thought to be absent in healthy adult skeletal muscle tissue [238], recent research demonstrated RAGE expression in muscle of young healthy subjects [239]. Further, RAGE overexpression has been documented in skeletal muscle of obese subjects [239] and streptozotocin (STZ)-induced diabetic mice [240]. Upon ligand biding, the cytoplasmic domain of RAGE interacts with several adaptor proteins (e.g., mammalian diaphanous 1 (mdia1/Diaph1) and TIR domain containing adaptor protein (TIRAP)) that connect RAGE with different pathways involved in cell stress signaling including JAK-STAT3, MAPKs (ERK1/2, p38, and JNK), IKK-NF-Κb, and AMPK-Akt [238]. Some of these pathways might result from RAGE-induced cytosolic ROS formation through activation of NADPH oxidases [241], which in turn might also promote mtROS generation (e.g., H_2_O_2_) [242].

Emerging evidence directly links AGE-RAGE signaling with skeletal muscle atrophy. Specifically, preclinical studies have linked AGE-RAGE signaling to AMPK-mediated downregulation of Akt, and subsequent MAFbx activation and atrophy in diabetic skeletal muscle [240]. Long-term administration of an AGE-rich diet to mice was shown to deteriorate skeletal muscle mass and performance, and suppress phosphorylation of mTORC1 downstream targets (S6K1) without impacting MHC isoforms [236]. In alignment, AGE treatment was reported to attenuate muscle load-induced hypertrophy and compromise muscle function in mice, which was accompanied by lower mTOR and S6K1 phosphorylation [243]. Other recent preclinical studies have observed that AGE treatment induces skeletal muscle atrophy and IR, leading to enhance Foxo1-MAFbx activity which was proposed to occur through ROS-induced ER stress [244]. Interestingly, circulating soluble isoforms of RAGE lacking domains needed for intracellular transduction might act as decoys for RAGE signaling, and reduced expression of these receptors has been reported in obesity and impaired glucose tolerance, which might contribute to the development of T2DM [245]. Altogether, glycated stress denoted by increased glucose-derived AGE levels might contribute to the loss of skeletal muscle mass and function shown in T2DM patients. Lastly, hyperglycemia might participate in skeletal muscle atrophy through different mechanisms. Particularly, hyperglycemia has been documented to exert direct catabolic effects on skeletal muscle through attenuated expression of the novel E3 ubiquitin ligase WWP1, which in turn prevents the degradation of zinc-finger transcription factor Krüppel-like factor 15 (KLF15), a transcription factor proposed to contribute to skeletal muscle atrophy potentially through FoxOs and atrogen [86, 246].

## Reciprocal impact of skeletal muscle atrophy and dysfunction on metabolic health

Skeletal muscle atrophy has negative effects in whole-body metabolism. For instance, impaired muscle mass results in decreased ability to store water, glycogen, and amino acids needed to regulate water and energy balance [14, 247]. In the context of metabolic disorders, impaired skeletal muscle plasticity can compromise functional capacity and the ability to conduct exercise. This is important since, together with healthy nutrition, sustained lifestyle interventions consisting of physical exercise programs are fundamental for treating obesity, preventing T2DM progression, and can potentially facilitate T2DM remission [248]. Most updated guidelines issued by expert organizations recommend aerobic exercise training programs supplemented with resistance training to delay or prevent the progression to T2DM, improve glycemic control, facilitate weight loss, and prevent cardiovascular complications [249, 250]. Particularly, the American College of Sports Medicine (ACSM) recommends adults with T2DM to engage in moderate (40–59% VO_2_ reserve (VO_2_R)) or intense (60–89% VO_2_R) aerobic exercise at least 3 days per week, with no more than 2 consecutive days between exercise bouts. This translates to 150–300 min/week of moderate aerobic activity or 75–150 min/week of vigorous exercise. Additionally, moderate (50–69% 1-repetition maximum (1RM)) or vigorous (70–85% 1RM) resistance training consisting of 10–15 repetitions per set and 1–3 repetitions per type of exercise is recommended 2–3 days per week, without consecutive days [250]. T2DM patients with comorbidities and older adults should attempt to conduct as much aerobic activity as possible, and maintain fitness and balance [250]. Naturally, these programs must be accompanied by sustainable eating plans based on current dietary guidelines for healthy diets [251], and tailored to the specific needs of the training program. Details on diabetes-focused nutritional guidance in the context of skeletal muscle atrophy and dysfunction have been previously reviewed elsewhere [51].

During exercise, increased energy demands of contracting muscles coupled with production of myokines and metabolic intermediates (e.g., lactate) are responsible for mediating interorgan communication, leading to beneficial metabolic adaptations including improved insulin sensitivity, which is critical to prevent the onset of T2DM (reviewed in ref. [252]). Regarding myocyte adaptations, mechanical loading due to muscle movement has profound myocellular repercussions that contribute to attenuate the loss of muscle mass and function associated with metabolic diseases through wide-ranging mechanisms. While a complete picture of all signaling events involved is not clear, exercise training potently activates AMPK, and increases cytoplasmic Ca^+2^ levels, thus modulating calcium-dependent signaling, mainly CaMKII, and downstream regulation of transcriptional activators and transcription factors (e.g., PGC-1α, PPAR-β, HDACs, MEF-2, etc.) [253]. Mechanotranducers and other stress-related signaling molecules (e.g., HIF-1α, MAPKs, ROS) are thought to participate in processes of skeletal muscle adaptation in response to different types of exercises [254]. For example, p38 MAPK can activate PGC-1 and Mef2 in response to aerobic exercise while ERK1/2 and JNK are involved in muscle hypertrophy through mTORC1 signaling and potential inhibition of myostatin resulting from resistance training [254]. However, contrary to sustained elevations seen in metabolic diseases, exercise elicits transient and pulsatile activation of these event cascades which associates to physiological effects leading to beneficial adaptations [253].

Overall, exercise induces adaptations that help counteract above-described deleterious effects of obesity and T2DM, being particularly important the effect of exercise on inducing GLUT4 expression in skeletal muscle (up to 100-fold compared to resting conditions) [255], which drives insulin-independent glucose uptake and attenuates increments in plasma glucose levels. Other key adaptations include promoted glycolytic-to-oxidative fiber type reprogramming, improved blood flow to the muscle, enhanced mitochondrial biogenesis, upregulated mitochondrial dynamics, and increased mitophagy flux particularly following endurance training [256, 257]. Resistance training, on the other hand, is the most powerful trigger of MPS through mTORC1 signaling, thus generating muscle mass when sufficient amino acid provisions are available [258]. Pulsatile release of myokines such as IL-6, particularly after aerobic exercise, regulates manifold processes in autocrine manner, including hypertrophy, lipolysis, and glucose disposal, among others [259]. Of note, while cross-communication between of muscle contraction to the gut microbiota is an expanding field of research, physical exercise modulates gastrointestinal transit times, this contributing to shaping the gut microbiota [260]. Impaired metabolism and decreased exercise tolerance resulting from skeletal muscle atrophy and dysfunction instigate a vice cycle in obese and T2DM patients which further aggravates the underlying metabolic conditions through the mechanisms herein discussed.

## Conclusion

Preventing or mitigating skeletal muscle atrophy and dysfunction has become a pivotal objective in combating obesity and curbing the progression to type 2 diabetes (T2DM). Chronic overnutrition, coupled with physical inactivity, precipitates a cascade of metabolic disturbances that undermine musculoskeletal plasticity, with hypertrophy of white adipose tissue emerging as a potential early indicator. Consequently, intramuscular lipid accumulation, skeletal muscle inflammation, mitochondrial dysfunction, oxidative stress, skeletal muscle fiber reprogramming, and hyperglycemia may contribute to muscle atrophy and dysfunction, with insulin resistance playing a central role due to its anticatabolic and permissive anabolic effects. Hyperglycemia is a major factor involved in the transition from obesity and T2DM, and sustained elevations of glucose levels in overt T2DM can further deteriorate skeletal muscle mass and quality in these patients through the production of advanced glycation end-products, leading to structural damage and activation of cellular receptors. Resulting muscle atrophy and dysfunction compromise the metabolic properties of the skeletal muscle, hinder the maintenance of healthy physical activity habits, and impede participation in exercise programs, thereby perpetuating a vicious cycle that exacerbates the underlying metabolic conditions.

## Data Availability

No datasets were generated or analysed during the current study.

## References

[1] Boutari C, DeMarsilis A, Mantzoros CS. Obesity and diabetes. Diabetes Res Clin Pract. 2023;202(110773). 10.1016/j.diabres.2023.110773. 10.1016/j.diabres.2023.110773 37356727

[2] Zhou B, Rayner AW, Gregg EW, Sheffer KE, Carrillo-Larco RM, Bennett JE, et al. Worldwide trends in diabetes prevalence and treatment from 1990 to 2022: a pooled analysis of 1108 population-representative studies with 141 million participants. Lancet. 2024;404(10467):2077–93. 39549716 10.1016/S0140-6736(24)02317-1 PMC7616842

[3] Phelps NH, Singleton RK, Zhou B, Heap RA, Mishra A, Bennett JE, et al. Worldwide trends in underweight and obesity from 1990 to 2022: a pooled analysis of 3663 population-representative studies with 222 million children, adolescents, and adults. Lancet. 2024;403(10431):1027–50. 38432237 10.1016/S0140-6736(23)02750-2 PMC7615769

[4] Bell JA, Kivimaki M, Hamer M. Metabolically healthy obesity and risk of incident type 2 diabetes: a meta-analysis of prospective cohort studies. Obes Rev. 2014;15(6):504–15. 24661566 10.1111/obr.12157 PMC4309497

[5] Abdullah A, Peeters A, de Courten M, Stoelwinder J. The magnitude of association between overweight and obesity and the risk of diabetes: A meta-analysis of prospective cohort studies. Diabetes Res Clin Pract. 2010;89(3):309–19. 10.1016/j.diabres.2010.04.012. 20493574 10.1016/j.diabres.2010.04.012

[6] Jayedi A, Soltani S, Motlagh SZ-t, Emadi A, Shahinfar H, Moosavi H, et al. Anthropometric and adiposity indicators and risk of type 2 diabetes: systematic review and dose-response meta-analysis of cohort studies. Bmj. 2022;376(e067516). 10.1136/bmj-2021-067516. 10.1136/bmj-2021-067516 PMC8764578 35042741

[7] Piché ME, Tchernof A, Després JP. Obesity phenotypes, diabetes, and cardiovascular diseases. Circ Res. 2020;126(11):1477–500. 10.1161/circresaha.120.316101. 32437302 10.1161/CIRCRESAHA.120.316101

[8] Michaelidou M, Pappachan JM, Jeeyavudeen MS. Management of diabesity: Current concepts. World J Diabetes. 2023;14(4):396–411. 10.4239/wjd.v14.i4.396. 37122433 10.4239/wjd.v14.i4.396 PMC10130896

[9] Frontera WR, Ochala J. Skeletal muscle: a brief review of structure and function. Calcif Tissue Int. 2015;96(3):183–95. 10.1007/s00223-014-9915-y. 25294644 10.1007/s00223-014-9915-y

[10] Murray B, Rosenbloom C. Fundamentals of glycogen metabolism for coaches and athletes. Nutr Rev. 2018;76(4):243–59. 29444266 10.1093/nutrit/nuy001 PMC6019055

[11] DeFronzo RA, Jacot E, Jequier E, Maeder E, Wahren J, Felber JP. The effect of insulin on the disposal of intravenous glucose: results from indirect calorimetry and hepatic and femoral venous catheterization. Diabetes. 1981;30(12):1000–7. 10.2337/diab.30.12.1000. 7030826 10.2337/diab.30.12.1000

[12] Zurlo F, Larson K, Bogardus C, Ravussin E. Skeletal muscle metabolism is a major determinant of resting energy expenditure. J Clin Investig. 1990;86(5):1423–7. 2243122 10.1172/JCI114857 PMC296885

[13] Pedersen BK, Febbraio MA. Muscles, exercise and obesity: skeletal muscle as a secretory organ. Nat Rev Endocrinol. 2012;8(8):457–65. 10.1038/nrendo.2012.49. 22473333 10.1038/nrendo.2012.49

[14] Argilés JM, Campos N, Lopez-Pedrosa JM, Rueda R, Rodriguez-Mañas L. Skeletal muscle regulates metabolism via interorgan crosstalk: roles in health and disease. J Am Med Dir Assoc. 2016;17(9):789–96. 27324808 10.1016/j.jamda.2016.04.019

[15] Pileggi CA, Parmar G, Harper M-E. The lifecycle of skeletal muscle mitochondria in obesity. Obes Rev. 2021;22(5):e13164. 10.1111/obr.13164. 33442950 10.1111/obr.13164

[16] Blüher M. Metabolically healthy obesity. Endocrine Reviews. 2020;41(3):bnaa004. 10.1210/endrev/bnaa004. 32128581 10.1210/endrev/bnaa004 PMC7098708

[17] Izzo A, Massimino E, Riccardi G, Della PG. A narrative review on sarcopenia in type 2 diabetes mellitus: prevalence and associated factors. Nutrients. 2021;13(1):183. 33435310 10.3390/nu13010183 PMC7826709

[18] Park SW, Goodpaster BH, Lee JS, Kuller LH, Boudreau R, De Rekeneire N, et al. Excessive loss of skeletal muscle mass in older adults with type 2 diabetes. Diabetes Care. 2009;32(11):1993–7. 19549734 10.2337/dc09-0264 PMC2768193

[19] Kim TN, Park MS, Yang SJ, Yoo HJ, Kang HJ, Song W, et al. Prevalence and determinant factors of sarcopenia in patients with type 2 diabetes: the Korean Sarcopenic Obesity Study (KSOS). Diabetes Care. 2010;33(7):1497–9. 20413515 10.2337/dc09-2310 PMC2890348

[20] Andersen H, Nielsen S, Mogensen CE, Jakobsen J. Muscle strength in type 2 diabetes. Diabetes. 2004;53(6):1543–8. 10.2337/diabetes.53.6.1543. 15161759 10.2337/diabetes.53.6.1543

[21] Anagnostis P, Gkekas NK, Achilla C, Pananastasiou G, Taouxidou P, Mitsiou M, et al. Type 2 diabetes mellitus is associated with increased risk of sarcopenia: a systematic review and meta-analysis. Calcif Tissue Int. 2020;107:453–63. 32772138 10.1007/s00223-020-00742-y

[22] Choi SJ, Files DC, Zhang T, Wang Z-M, Messi ML, Gregory H, et al. Intramyocellular lipid and impaired myofiber contraction in normal weight and obese older adults. J Gerontol: Ser A. 2015;71(4):557–64. 10.1093/gerona/glv169. 10.1093/gerona/glv169 PMC5014190 26405061

[23] Smeuninx B, McKendry J, Wilson D, Martin U, Breen L. Age-related anabolic resistance of myofibrillar protein synthesis is exacerbated in obese inactive individuals. J Clin Endocrinol Metab. 2017;102(9):3535–45. 10.1210/jc.2017-00869. 28911148 10.1210/jc.2017-00869 PMC5587073

[24] Hulens M, Vansant G, Lysens R, Claessens AL, Muls E, Brumagne S. Study of differences in peripheral muscle strength of lean versus obese women: an allometric approach. Int J Obes. 2001;25(5):676–81. 10.1038/sj.ijo.0801560. 10.1038/sj.ijo.0801560 11360150

[25] Tomlinson DJ, Erskine RM, Morse CI, Winwood K, Onambélé-Pearson G. The impact of obesity on skeletal muscle strength and structure through adolescence to old age. Biogerontology. 2016;17(3):467–83. 10.1007/s10522-015-9626-4. 26667010 10.1007/s10522-015-9626-4 PMC4889641

[26] Tomlinson DJ, Erskine RM, Morse CI, Winwood K, Onambélé-Pearson GL. Combined effects of body composition and ageing on joint torque, muscle activation and co-contraction in sedentary women. Age (Dordr). 2014;36(3):9652. 10.1007/s11357-014-9652-1. 24744050 10.1007/s11357-014-9652-1 PMC4082607

[27] MÅrin P, Andersson B, Krotkiewski M, Björntorp P. Muscle fiber composition and capillary density in women and men with NIDDM. Diabetes Care. 1994;17(5):382–6. 8062604 10.2337/diacare.17.5.382

[28] Oberbach A, Bossenz Y, Lehmann S, Niebauer J, Adams V, Paschke R, et al. Altered fiber distribution and fiber-specific glycolytic and oxidative enzyme activity in skeletal muscle of patients with type 2 diabetes. Diabetes Care. 2006;29(4):895–900. 16567834 10.2337/diacare.29.04.06.dc05-1854

[29] Gaster M, Staehr P, Beck-Nielsen H, Schrøder HD, Handberg A. GLUT4 is reduced in slow muscle fibers of type 2 diabetic patients: is insulin resistance in type 2 diabetes a slow, type 1 fiber disease? Diabetes. 2001;50(6):1324–9. 11375332 10.2337/diabetes.50.6.1324

[30] Frankenberg NT, Mason SA, Wadley GD, Murphy RM. Skeletal muscle cell-specific differences in type 2 diabetes. Cell Mol Life Sci. 2022;79(5):256. 35460430 10.1007/s00018-022-04265-7 PMC9035013

[31] Helge JW, Fraser AM, Kriketos AD, Jenkins AB, Calvert GD, Ayre KJ, et al. Interrelationships between muscle fibre type, substrate oxidation and body fat. Int J Obes. 1999;23(9):986–91. 10.1038/sj.ijo.0801030. 10.1038/sj.ijo.0801030 10490806

[32] Kriketos AD, Pan DA, Lillioja S, Cooney GJ, Baur LA, Milner MR, et al. Interrelationships between muscle morphology, insulin action, and adiposity. Am J Physiol. 1996;270(6 Pt 2):R1332–9. 10.1152/ajpregu.1996.270.6.R1332. 8764301 10.1152/ajpregu.1996.270.6.R1332

[33] Tanner CJ, Barakat HA, Dohm GL, Pories WJ, MacDonald KG, Cunningham PR, et al. Muscle fiber type is associated with obesity and weight loss. Am J Physiol Endocrinol Metab. 2002;282(6):E1191–6. 10.1152/ajpendo.00416.2001. 12006347 10.1152/ajpendo.00416.2001

[34] Zierath J, He L, Guma A, Wahlström EO, Klip A, Wallberg-Henriksson H. Insulin action on glucose transport and plasma membrane GLUT4 content in skeletal muscle from patients with NIDDM. Diabetologia. 1996;39:1180–9. 8897005 10.1007/BF02658504

[35] Groen BB, Hamer HM, Snijders T, Van Kranenburg J, Frijns D, Vink H, et al. Skeletal muscle capillary density and microvascular function are compromised with aging and type 2 diabetes. J Appl Physiol. 2014;116(8):998–1005. 24577061 10.1152/japplphysiol.00919.2013

[36] Benedict KF, Coffin GS, Barrett EJ, Skalak TC. Hemodynamic systems analysis of capillary network remodeling during the progression of type 2 diabetes. Microcirculation. 2011;18(1):63–73. 21166927 10.1111/j.1549-8719.2010.00069.x

[37] de Jongh RT, Serné EH, Ijzerman RG, de Vries G, Stehouwer CDA. Impaired microvascular function in obesity. Circulation. 2004;109(21):2529–35. 10.1161/01.CIR.0000129772.26647.6F. 15136505 10.1161/01.CIR.0000129772.26647.6F

[38] Vinet A, Karpoff L, Walther G, Startun A, Obert P, Goret L, et al. Vascular reactivity at rest and during exercise in middle-aged obese men: effects of short-term, low-intensity, exercise training. Int J Obes. 2011;35(6):820–8. 10.1038/ijo.2010.206. 10.1038/ijo.2010.206 20877288

[39] Bosma M. Lipid droplet dynamics in skeletal muscle. Exp Cell Res. 2016;340(2):180–6. 10.1016/j.yexcr.2015.10.023. 26515552 10.1016/j.yexcr.2015.10.023

[40] de Almeida ME, Nielsen J, Petersen MH, Wentorf EK, Pedersen NB, Jensen K, et al. Altered intramuscular network of lipid droplets and mitochondria in type 2 diabetes. Am J Physiol-Cell Physiol. 2023;324(1):C39–57. 36409174 10.1152/ajpcell.00470.2022

[41] Daemen S, Gemmink A, Brouwers B, Meex RC, Huntjens PR, Schaart G, et al. Distinct lipid droplet characteristics and distribution unmask the apparent contradiction of the athlete’s paradox. Molecular metabolism. 2018;17:71–81. 30174227 10.1016/j.molmet.2018.08.004 PMC6197504

[42] van Herpen NA, Schrauwen-Hinderling VB. Lipid accumulation in non-adipose tissue and lipotoxicity. Physiol Behav. 2008;94(2):231–41. 10.1016/j.physbeh.2007.11.049. 18222498 10.1016/j.physbeh.2007.11.049

[43] Engin AB. What is lipotoxicity? Obesity and lipotoxicity. 2017. 197–220. 10.1007/978-3-319-48382-5_8.

[44] Berria R, Wang L, Richardson DK, Finlayson J, Belfort R, Pratipanawatr T, et al. Increased collagen content in insulin-resistant skeletal muscle. Am J Physiol-Endocrinol Metab. 2006;290(3):E560–5. 10.1152/ajpendo.00202.2005. 16249255 10.1152/ajpendo.00202.2005

[45] Tam CS, Covington JD, Bajpeyi S, Tchoukalova Y, Burk D, Johannsen DL, et al. Weight gain reveals dramatic increases in skeletal muscle extracellular matrix remodeling. J Clin Endocrinol Metab. 2014;99(5):1749–57. 10.1210/jc.2013-4381. 24601694 10.1210/jc.2013-4381 PMC4010691

[46] Nesti L, Pugliese NR, Sciuto P, Natali A. Type 2 diabetes and reduced exercise tolerance: a review of the literature through an integrated physiology approach. Cardiovasc Diabetol. 2020;19(1):134. 10.1186/s12933-020-01109-1. 32891175 10.1186/s12933-020-01109-1 PMC7487838

[47] Petersen MC, Smith GI, Palacios HH, Farabi SS, Yoshino M, Yoshino J, et al. Cardiometabolic characteristics of people with metabolically healthy and unhealthy obesity. Cell Metab. 2024;36(4):745-61.e5. 10.1016/j.cmet.2024.03.002. 38569471 10.1016/j.cmet.2024.03.002 PMC11025492

[48] Smith GI, Mittendorfer B, Klein S. Metabolically healthy obesity: facts and fantasies. J Clin Investig. 2019;129(10):3978–89. 31524630 10.1172/JCI129186 PMC6763224

[49] Baum T, Cordes C, Dieckmeyer M, Ruschke S, Franz D, Hauner H, et al. MR-based assessment of body fat distribution and characteristics. Eur J Radiol. 2016;85(8):1512–8. 10.1016/j.ejrad.2016.02.013. 26905521 10.1016/j.ejrad.2016.02.013

[50] Schulze MB, Stefan N. Metabolically healthy obesity: from epidemiology and mechanisms to clinical implications. Nat Rev Endocrinol. 2024;20(11):633–46. 38937638 10.1038/s41574-024-01008-5

[51] Lopez-Pedrosa JM, Camprubi-Robles M, Guzman-Rolo G, Lopez-Gonzalez A, Garcia-Almeida JM, Sanz-Paris A, et al. The vicious cycle of type 2 diabetes mellitus and skeletal muscle atrophy: clinical, biochemical, and nutritional bases. Nutrients. 2024;16(1):172. 38202001 10.3390/nu16010172 PMC10780454

[52] James DE, Stöckli J, Birnbaum MJ. The aetiology and molecular landscape of insulin resistance. Nat Rev Mol Cell Biol. 2021;22(11):751–71. 10.1038/s41580-021-00390-6. 34285405 10.1038/s41580-021-00390-6

[53] Klöting N, Fasshauer M, Dietrich A, Kovacs P, Schön MR, Kern M, et al. Insulin-sensitive obesity. Am J Physiol-Endocrinol Metab. 2010;299(3):E506–15. 20570822 10.1152/ajpendo.00586.2009

[54] Gustafson B, Hedjazifar S, Gogg S, Hammarstedt A, Smith U. Insulin resistance and impaired adipogenesis. Trends Endocrinol Metab. 2015;26(4):193–200. 25703677 10.1016/j.tem.2015.01.006

[55] Hosogai N, Fukuhara A, Oshima K, Miyata Y, Tanaka S, Segawa K, et al. Adipose tissue hypoxia in obesity and its impact on adipocytokine dysregulation. Diabetes. 2007;56(4):901–11. 10.2337/db06-0911. 17395738 10.2337/db06-0911

[56] Caselli C. Role of adiponectin system in insulin resistance. Mol Genet Metab. 2014;113(3):155–60. 10.1016/j.ymgme.2014.09.003. 25242063 10.1016/j.ymgme.2014.09.003

[57] Castela I, Morais J, Barreiros-Mota I, Silvestre MP, Marques C, Rodrigues C, et al. Decreased adiponectin/leptin ratio relates to insulin resistance in adults with obesity. Am J Physiol-Endocrinol Metab. 2023;324(2):E115–9. 10.1152/ajpendo.00273.2022. 36351292 10.1152/ajpendo.00273.2022

[58] Hotamisligil GS, Shargill NS, Spiegelman BM. Adipose expression of tumor necrosis factor-α: direct role in obesity-linked insulin resistance. Science. 1993;259(5091):87–91. 10.1126/science.7678183. 7678183 10.1126/science.7678183

[59] Gutierrez-Rodelo C, Arellano-Plancarte A, Hernandez-Aranda J, Landa-Galvan HV, Parra-Mercado GK, Moreno-Licona NJ, et al. Angiotensin II inhibits insulin receptor signaling in adipose cells. Int J Mol Sci. 2022;23(11):6048. 35682723 10.3390/ijms23116048 PMC9181642

[60] Kwon H, Pessin JE. Adipokines mediate inflammation and insulin resistance. Front Endocrinol. 2013;4:71. 10.3389/fendo.2013.00071 PMC3679475 23781214

[61] Khan IM, Perrard XY, Brunner G, Lui H, Sparks LM, Smith SR, et al. Intermuscular and perimuscular fat expansion in obesity correlates with skeletal muscle T cell and macrophage infiltration and insulin resistance. Int J Obes. 2015;39(11):1607–18. 10.1038/ijo.2015.104. 10.1038/ijo.2015.104 PMC5007876 26041698

[62] Stephens JM, Lee J, Pilch PF. Tumor necrosis factor-α-induced insulin resistance in 3T3-L1 adipocytes is accompanied by a loss of insulin receptor substrate-1 and GLUT4 expression without a loss of insulin receptor-mediated signal transduction*. J Biol Chem. 1997;272(2):971–6. 10.1074/jbc.272.2.971. 8995390 10.1074/jbc.272.2.971

[63] Frigolet ME, Torres N, Tovar AR. The renin–angiotensin system in adipose tissue and its metabolic consequences during obesity. J Nutr Biochem. 2013;24(12):2003–15. 10.1016/j.jnutbio.2013.07.002. 24120291 10.1016/j.jnutbio.2013.07.002

[64] Rasmussen MS, Lihn AS, Pedersen SB, Bruun JM, Rasmussen M, Richelsen B. Adiponectin receptors in human adipose tissue: effects of obesity, weight loss, and fat depots. Obesity. 2006;14(1):28–35. 10.1038/oby.2006.5. 16493120 10.1038/oby.2006.5

[65] Hoehn KL, Salmon AB, Hohnen-Behrens C, Turner N, Hoy AJ, Maghzal GJ, et al. Insulin resistance is a cellular antioxidant defense mechanism. Proc Natl Acad Sci USA. 2009;106(42):17787–92. 10.1073/pnas.0902380106. 19805130 10.1073/pnas.0902380106 PMC2764908

[66] Jiao P, Ma J, Feng B, Zhang H, Alan-Diehl J, Eugene-Chin Y, et al. FFA-induced adipocyte inflammation and insulin resistance: involvement of ER stress and IKKβ pathways. Obesity. 2011;19(3):483–91. 10.1038/oby.2010.200. 20829802 10.1038/oby.2010.200

[67] Morigny P, Houssier M, Mouisel E, Langin D. Adipocyte lipolysis and insulin resistance. Biochimie. 2016;125:259–66. 10.1016/j.biochi.2015.10.024. 26542285 10.1016/j.biochi.2015.10.024

[68] Drolet R, Richard C, Sniderman AD, Mailloux J, Fortier M, Huot C, et al. Hypertrophy and hyperplasia of abdominal adipose tissues in women. Int J Obes. 2008;32(2):283–91. 10.1038/sj.ijo.0803708. 10.1038/sj.ijo.0803708 17726433

[69] Baldini F, Fabbri R, Eberhagen C, Voci A, Portincasa P, Zischka H, et al. Adipocyte hypertrophy parallels alterations of mitochondrial status in a cell model for adipose tissue dysfunction in obesity. Life Sci. 2021;265:118812. 10.1016/j.lfs.2020.118812. 33278396 10.1016/j.lfs.2020.118812

[70] Weyer C, Foley JE, Bogardus C, Tataranni PA, Pratley RE. Enlarged subcutaneous abdominal adipocyte size, but not obesity itself, predicts Type II diabetes independent of insulin resistance. Diabetologia. 2000;43(12):1498–506. 10.1007/s001250051560. 11151758 10.1007/s001250051560

[71] Giordano A, Murano I, Mondini E, Perugini J, Smorlesi A, Severi I, et al. Obese adipocytes show ultrastructural features of stressed cells and die of pyroptosis. J Lipid Res. 2013;54(9):2423–36. 10.1194/jlr.M038638. 23836106 10.1194/jlr.M038638 PMC3735940

[72] Verboven K, Wouters K, Gaens K, Hansen D, Bijnen M, Wetzels S, et al. Abdominal subcutaneous and visceral adipocyte size, lipolysis and inflammation relate to insulin resistance in male obese humans. Sci Rep. 2018;8(1):4677. 10.1038/s41598-018-22962-x. 29549282 10.1038/s41598-018-22962-x PMC5856747

[73] Ye J, Gao Z, Yin J, He Q. Hypoxia is a potential risk factor for chronic inflammation and adiponectin reduction in adipose tissue of ob/ob and dietary obese mice. Am J Physiol-Endocrinol Metab. 2007;293(4):E1118–28. 10.1152/ajpendo.00435.2007. 17666485 10.1152/ajpendo.00435.2007

[74] Politis-Barber V, Brunetta HS, Paglialunga S, Petrick HL, Holloway GP. Long-term, high-fat feeding exacerbates short-term increases in adipose mitochondrial reactive oxygen species, without impairing mitochondrial respiration. Am J Physiol-Endocrinol Metab. 2020;319(2):E376–87. 32543945 10.1152/ajpendo.00028.2020 PMC7473917

[75] Lee YS, Kim J-w, Osborne O, Sasik R, Schenk S, Chen A, et al. Increased adipocyte O2 consumption triggers HIF-1α, causing inflammation and insulin resistance in obesity. Cell. 2014;157(6):1339–52. 24906151 10.1016/j.cell.2014.05.012 PMC4114226

[76] Tanaka M, Ikeda K, Suganami T, Komiya C, Ochi K, Shirakawa I, et al. Macrophage-inducible C-type lectin underlies obesity-induced adipose tissue fibrosis. Nat Commun. 2014;5(1):4982. 10.1038/ncomms5982. 25236782 10.1038/ncomms5982

[77] Ji Y, Li M, Chang M, Liu R, Qiu J, Wang K, et al. Inflammation: roles in skeletal muscle atrophy. Antioxidants. 2022;11(9):1686. 36139760 10.3390/antiox11091686 PMC9495679

[78] Mosser RE, Maulis MF, Moullé VS, Dunn JC, Carboneau BA, Arasi K, et al. High-fat diet-induced β-cell proliferation occurs prior to insulin resistance in C57Bl/6J male mice. Am J Physiol-Endocrinol Metab. 2015;308(7):E573–82. 25628421 10.1152/ajpendo.00460.2014 PMC4385873

[79] van Vliet S, Koh H-CE, Patterson BW, Yoshino M, LaForest R, Gropler RJ, et al. Obesity is associated with increased basal and postprandial β-cell insulin secretion even in the absence of insulin resistance. Diabetes. 2020;69(10):2112–9. 32651241 10.2337/db20-0377 PMC7506835

[80] Mir SU, George NM, Zahoor L, Harms R, Guinn Z, Sarvetnick NE. Inhibition of autophagic turnover in β-cells by fatty acids and glucose leads to apoptotic cell death. J Biol Chem. 2015;290(10):6071–85. 25548282 10.1074/jbc.M114.605345 PMC4358249

[81] Cerf ME. Beta cell dysfunction and insulin resistance. Front Endocrinol. 2013;4:37. 10.3389/fendo.2013.00037 PMC3608918 23542897

[82] Gastaldelli A, Cusi K, Pettiti M, Hardies J, Miyazaki Y, Berria R, et al. Relationship between hepatic/visceral fat and hepatic insulin resistance in nondiabetic and type 2 diabetic subjects. Gastroenterology. 2007;133(2):496–506. 10.1053/j.gastro.2007.04.068. 17681171 10.1053/j.gastro.2007.04.068

[83] Fu Z, R Gilbert E, Liu D. Regulation of insulin synthesis and secretion and pancreatic Beta-cell dysfunction in diabetes. Curr Diabetes Rev. 2013;9(1):25–53. 22974359 PMC3934755

[84] Miki A, Ricordi C, Sakuma Y, Yamamoto T, Misawa R, Mita A, et al. Divergent antioxidant capacity of human islet cell subsets: A potential cause of beta-cell vulnerability in diabetes and islet transplantation. PLoS ONE. 2018;13(5):e0196570. 10.1371/journal.pone.0196570. 29723228 10.1371/journal.pone.0196570 PMC5933778

[85] Gerber PA, Rutter GA. The role of oxidative stress and hypoxia in pancreatic beta-cell dysfunction in diabetes mellitus. Antioxid Redox Signal. 2017;26(10):501–18. 27225690 10.1089/ars.2016.6755 PMC5372767

[86] Hirata Y, Nomura K, Senga Y, Okada Y, Kobayashi K, Okamoto S, et al. Hyperglycemia induces skeletal muscle atrophy via a WWP1/KLF15 axis. JCI Insight. 2019;4(4):e124952. 10.1172/jci.insight.124952. 30830866 10.1172/jci.insight.124952 PMC6478420

[87] Suzuki A, Yabu A, Nakamura H. Advanced glycation end products in musculoskeletal system and disorders. Methods. 2022;203:179–86. 10.1016/j.ymeth.2020.09.012. 32987130 10.1016/j.ymeth.2020.09.012

[88] Kashyap SR, Roman LJ, Lamont J, Masters BSS, Bajaj M, Suraamornkul S, et al. Insulin resistance is associated with impaired nitric oxide synthase activity in skeletal muscle of type 2 diabetic subjects. J Clin Endocrinol Metab. 2005;90(2):1100–5. 10.1210/jc.2004-0745. 15562034 10.1210/jc.2004-0745

[89] Jenkins HN, Rivera-Gonzalez O, Gibert Y, Speed JS. Endothelin-1 in the pathophysiology of obesity and insulin resistance. Obes Rev. 2020;21(12):e13086. 32627269 10.1111/obr.13086 PMC7669671

[90] Liu S-Y, Chen L-K, Jhong Y-T, Chen C-W, Hsiao L-E, Ku H-C, et al. Endothelin-1 impairs skeletal muscle myogenesis and development via ETB receptors and p38 MAPK signaling pathway. Clin Sci. 2024;138(12):711–23. 10.1042/cs20240341. 10.1042/CS20240341 38804865

[91] Banks NF, Rogers EM, Church DD, Ferrando AA, Jenkins ND. The contributory role of vascular health in age-related anabolic resistance. J Cachexia Sarcopenia Muscle. 2022;13(1):114–27. 10.1002/jcsm.12898. 34951146 10.1002/jcsm.12898 PMC8818606

[92] Bleau C, Karelis AD, St-Pierre DH, Lamontagne L. Crosstalk between intestinal microbiota, adipose tissue and skeletal muscle as an early event in systemic low-grade inflammation and the development of obesity and diabetes. Diabetes Metab Res Rev. 2015;31(6):545–61. 10.1002/dmrr.2617. 25352002 10.1002/dmrr.2617

[93] Cox AJ, Zhang P, Bowden DW, Devereaux B, Davoren PM, Cripps AW, et al. Increased intestinal permeability as a risk factor for type 2 diabetes. Diabetes Metab. 2017;43(2):163–6. 10.1016/j.diabet.2016.09.004. 27745826 10.1016/j.diabet.2016.09.004

[94] Teixeira TFS, Souza NCS, Chiarello PG, Franceschini SCC, Bressan J, Ferreira CLLF, et al. Intestinal permeability parameters in obese patients are correlated with metabolic syndrome risk factors. Clin Nutr. 2012;31(5):735–40. 10.1016/j.clnu.2012.02.009. 22444236 10.1016/j.clnu.2012.02.009

[95] Cani PD, Amar J, Iglesias MA, Poggi M, Knauf C, Bastelica D, et al. Metabolic endotoxemia initiates obesity and insulin resistance. Diabetes. 2007;56(7):1761–72. 10.2337/db06-1491. 17456850 10.2337/db06-1491

[96] Fang W-Y, Tseng Y-T, Lee T-Y, Fu Y-C, Chang W-H, Lo W-W, et al. Triptolide prevents LPS-induced skeletal muscle atrophy via inhibiting NF-κB/TNF-α and regulating protein synthesis/degradation pathway. Br J Pharmacol. 2021;178(15):2998–3016. 10.1111/bph.15472. 33788266 10.1111/bph.15472

[97] Scheithauer TP, Rampanelli E, Nieuwdorp M, Vallance BA, Verchere CB, Van Raalte DH, et al. Gut microbiota as a trigger for metabolic inflammation in obesity and type 2 diabetes. Front Immunol. 2020;11:571731. 33178196 10.3389/fimmu.2020.571731 PMC7596417

[98] Martin-Gallausiaux C, Marinelli L, Blottière HM, Larraufie P, Lapaque N. SCFA: mechanisms and functional importance in the gut. Proc Nutr Soc. 2021;80(1):37–49. 32238208 10.1017/S0029665120006916

[99] Fragala MS, Kenny AM, Kuchel GA. Muscle quality in aging: a multi-dimensional approach to muscle functioning with applications for treatment. Sports Med. 2015;45:641–58. 25655372 10.1007/s40279-015-0305-z

[100] Correa-de-Araujo R, Harris-Love MO, Miljkovic I, Fragala MS, Anthony BW, Manini TM. The need for standardized assessment of muscle quality in skeletal muscle function deficit and other aging-related muscle dysfunctions: a symposium report. Front Physiol. 2017;8:87. 10.3389/fphys.2017.00087. 28261109 10.3389/fphys.2017.00087 PMC5310167

[101] Abdulla H, Smith K, Atherton PJ, Idris I. Role of insulin in the regulation of human skeletal muscle protein synthesis and breakdown: a systematic review and meta-analysis. Diabetologia. 2016;59(1):44–55. 10.1007/s00125-015-3751-0. 26404065 10.1007/s00125-015-3751-0

[102] Fujita S, Glynn EL, Timmerman KL, Rasmussen BB, Volpi E. Supraphysiological hyperinsulinaemia is necessary to stimulate skeletal muscle protein anabolism in older adults: evidence of a true age-related insulin resistance of muscle protein metabolism. Diabetologia. 2009;52(9):1889–98. 10.1007/s00125-009-1430-8. 19588121 10.1007/s00125-009-1430-8 PMC2843438

[103] Timmerman KL, Lee JL, Dreyer HC, Dhanani S, Glynn EL, Fry CS, et al. Insulin stimulates human skeletal muscle protein synthesis via an indirect mechanism involving endothelial-dependent vasodilation and mammalian target of rapamycin complex 1 signaling. J Clin Endocrinol Metab. 2010;95(8):3848–57. 10.1210/jc.2009-2696. 20484484 10.1210/jc.2009-2696 PMC2913031

[104] Aslesh T, Al-aghbari A, Yokota T. Assessing the role of aquaporin 4 in skeletal muscle function. Int J Mol Sci. 2023;24(2):1489. 36675000 10.3390/ijms24021489 PMC9865462

[105] Chung SW, Kim J-Y, Yoon JP, Suh DW, Yeo WJ, Lee Y-S. Atrogin1-induced loss of aquaporin 4 in myocytes leads to skeletal muscle atrophy. Sci Rep. 2020;10(1):14189. 10.1038/s41598-020-71167-8. 32843684 10.1038/s41598-020-71167-8 PMC7447774

[106] Khalid M, Alkaabi J, Khan MAB, Adem A. Insulin signal transduction perturbations in insulin resistance. Int J Mol Sci. 2021;22(16):8590. 34445300 10.3390/ijms22168590 PMC8395322

[107] Luo J, Sobkiw CL, Hirshman MF, Logsdon MN, Li TQ, Goodyear LJ, et al. Loss of class IA PI3K signaling in muscle leads to impaired muscle growth, insulin response, and hyperlipidemia. Cell Metab. 2006;3(5):355–66. 10.1016/j.cmet.2006.04.003. 16679293 10.1016/j.cmet.2006.04.003

[108] Hargett SR, Walker NN, Keller SR. Rab GAPs AS160 and Tbc1d1 play nonredundant roles in the regulation of glucose and energy homeostasis in mice. Am J Physiol-Endocrinol Metab. 2016;310(4):E276–88. 10.1152/ajpendo.00342.2015. 26625902 10.1152/ajpendo.00342.2015 PMC4888528

[109] Sylow L, Tokarz VL, Richter EA, Klip A. The many actions of insulin in skeletal muscle, the paramount tissue determining glycemia. Cell Metab. 2021;33(4):758–80. 10.1016/j.cmet.2021.03.020. 33826918 10.1016/j.cmet.2021.03.020

[110] Goul C, Peruzzo R, Zoncu R. The molecular basis of nutrient sensing and signalling by mTORC1 in metabolism regulation and disease. Nat Rev Mol Cell Biol. 2023;24(12):857–75. 10.1038/s41580-023-00641-8. 37612414 10.1038/s41580-023-00641-8

[111] Mammucari C, Schiaffino S, Sandri M. Downstream of Akt: FoxO3 and mTOR in the regulation of autophagy in skeletal muscle. Autophagy. 2008;4(4):524–6. 10.4161/auto.5905. 18367868 10.4161/auto.5905

[112] Jaiswal N, Gavin M, Loro E, Sostre-Colón J, Roberson PA, Uehara K, et al. AKT controls protein synthesis and oxidative metabolism via combined mTORC1 and FOXO1 signalling to govern muscle physiology. J Cachexia Sarcopenia Muscle. 2022;13(1):495–514. 10.1002/jcsm.12846. 34751006 10.1002/jcsm.12846 PMC8818654

[113] Bodine SC, Baehr LM. Skeletal muscle atrophy and the E3 ubiquitin ligases MuRF1 and MAFbx/atrogin-1. Am J Physiol Endocrinol Metab. 2014;307(6):E469–84. 10.1152/ajpendo.00204.2014. 25096180 10.1152/ajpendo.00204.2014 PMC4166716

[114] Clavel S, Siffroi-Fernandez S, Coldefy AS, Boulukos K, Pisani DF, Dérijard B. Regulation of the intracellular localization of Foxo3a by stress-activated protein kinase signaling pathways in skeletal muscle cells. Mol Cell Biol. 2010;30(2):470–80. 19917721 10.1128/MCB.00666-09 PMC2798458

[115] Klotz L-O, Sánchez-Ramos C, Prieto-Arroyo I, Urbánek P, Steinbrenner H, Monsalve M. Redox regulation of FoxO transcription factors. Redox Biol. 2015;6:51–72. 10.1016/j.redox.2015.06.019. 26184557 10.1016/j.redox.2015.06.019 PMC4511623

[116] Schönfeld P, Wojtczak L. Short-and medium-chain fatty acids in energy metabolism: the cellular perspective. J Lipid Res. 2016;57(6):943–54. 27080715 10.1194/jlr.R067629 PMC4878196

[117] Sun J, Su Y, Chen J, Qin D, Xu Y, Chu H, et al. Differential roles of CD36 in regulating muscle insulin response depend on palmitic acid load. Biomedicines. 2023;11(3):729. 36979708 10.3390/biomedicines11030729 PMC10045334

[118] Bonen A, Parolin ML, Steinberg GR, Calles-Escandon J, Tandon NN, Glatz JFC, et al. Triacylglycerol accumulation in human obesity and type 2 diabetes is associated with increased rates of skeletal muscle fatty acid transport and increased sarcolemmal FAT/CD36. FASEB J. 2004;18(10):1144–6. 10.1096/fj.03-1065fje. 15132977 10.1096/fj.03-1065fje

[119] Clarke DC, Miskovic D, Han X-X, Calles-Escandon J, Glatz JFC, Luiken JJFP, et al. Overexpression of membrane-associated fatty acid binding protein (FABPpm) *in vivo* increases fatty acid sarcolemmal transport and metabolism. Physiol Genomics. 2004;17(1):31–7. 10.1152/physiolgenomics.00190.2003. 14694205 10.1152/physiolgenomics.00190.2003

[120] Glatz JFC, Luiken JJFP, Bonen A. Membrane fatty acid transporters as regulators of lipid metabolism: implications for metabolic disease. Physiol Rev. 2010;90(1):367–417. 10.1152/physrev.00003.2009. 20086080 10.1152/physrev.00003.2009

[121] Roepstorff C, Wulff Helge J, Vistisen B, Kiens B. Studies of plasma membrane fatty acid-binding protein and other lipid-binding proteins in human skeletal muscle. Proc Nutr Soc. 2007;63(2):239–44. 10.1079/PNS2004332. 10.1079/PNS2004332 15294037

[122] Kawaguchi M, Tamura Y, Kakehi S, Takeno K, Sakurai Y, Watanabe T, et al. Association between expression of FABPpm in skeletal muscle and insulin sensitivity in intramyocellular lipid-accumulated nonobese men. J Clin Endocrinol Metab. 2014;99(9):3343–52. 10.1210/jc.2014-1896. 24937540 10.1210/jc.2014-1896

[123] Lundsgaard A-M, Fritzen AM, Kiens B. Molecular regulation of fatty acid oxidation in skeletal muscle during aerobic exercise. Trends Endocrinol Metab. 2018;29(1):18–30. 29221849 10.1016/j.tem.2017.10.011

[124] Kelley DE, He J, Menshikova EV, Ritov VB. Dysfunction of mitochondria in human skeletal muscle in type 2 diabetes. Diabetes. 2002;51(10):2944–50. 10.2337/diabetes.51.10.2944. 12351431 10.2337/diabetes.51.10.2944

[125] Pileggi CA, Parmar G, Harper ME. The lifecycle of skeletal muscle mitochondria in obesity. Obes Rev. 2021;22(5):e13164. 33442950 10.1111/obr.13164

[126] Timmers S, Schrauwen P, de Vogel J. Muscular diacylglycerol metabolism and insulin resistance. Physiol Behav. 2008;94(2):242–51. 18207474 10.1016/j.physbeh.2007.12.002

[127] Szendroedi J, Yoshimura T, Phielix E, Koliaki C, Marcucci M, Zhang D, et al. Role of diacylglycerol activation of PKCθ in lipid-induced muscle insulin resistance in humans. Proc Natl Acad Sci USA. 2014;111(26):9597–602. 10.1073/pnas.1409229111. 24979806 10.1073/pnas.1409229111 PMC4084449

[128] Jayasinghe SU, Tankeu AT, Amati F. Reassessing the role of diacylglycerols in insulin resistance. Trends Endocrinol Metab. 2019;30(9):618–35. 10.1016/j.tem.2019.06.005. 31375395 10.1016/j.tem.2019.06.005

[129] Gaspar RC, Lyu K, Hubbard BT, Leitner BP, Luukkonen PK, Hirabara SM, et al. Distinct subcellular localisation of intramyocellular lipids and reduced PKCε/PKCθ activity preserve muscle insulin sensitivity in exercise-trained mice. Diabetologia. 2023;66(3):567–78. 10.1007/s00125-022-05838-8. 36456864 10.1007/s00125-022-05838-8 PMC11194860

[130] Li Y, Soos TJ, Li X, Wu J, DeGennaro M, Sun X, et al. Protein kinase C θ inhibits insulin signaling by phosphorylating IRS1 at Ser1101. J Biol Chem. 2004;279(44):45304–7. 15364919 10.1074/jbc.C400186200

[131] Jollet M, Tramontana F, Jiang LQ, Borg ML, Savikj M, Kuefner MS, et al. Diacylglycerol kinase delta overexpression improves glucose clearance and protects against the development of obesity. Metabolism. 2024;158:155939. 10.1016/j.metabol.2024.155939. 38843995 10.1016/j.metabol.2024.155939

[132] Wada Y, Sakiyama S, Sakai H, Sakane F. Myristic acid enhances diacylglycerol kinase δ-dependent glucose uptake in myotubes. Lipids. 2016;51:897–903. 27206979 10.1007/s11745-016-4162-9

[133] Turpin-Nolan SM, Hammerschmidt P, Chen W, Jais A, Timper K, Awazawa M, et al. CerS1-derived C18: 0 ceramide in skeletal muscle promotes obesity-induced insulin resistance. Cell Rep. 2019;26(1):1-10. e7. 30605666 10.1016/j.celrep.2018.12.031

[134] Bandet C, Tan-Chen S, Bourron O, Le Stunff H, Hajduch E. Sphingolipid metabolism: new insight into ceramide-induced lipotoxicity in muscle cells. Int J Mol Sci. 2019;20:479. 10.3390/ijms20030479. 30678043 10.3390/ijms20030479 PMC6387241

[135] Bandet CL, Mahfouz R, Véret J, Sotiropoulos A, Poirier M, Giussani P, et al. Ceramide transporter CERT is involved in muscle insulin signaling defects under lipotoxic conditions. Diabetes. 2018;67(7):1258–71. 10.2337/db17-0901. 29759974 10.2337/db17-0901

[136] Powell DJ, Turban S, Gray A, Hajduch E, Hundal HS. Intracellular ceramide synthesis and protein kinase Czeta activation play an essential role in palmitate-induced insulin resistance in rat L6 skeletal muscle cells. Biochem J. 2004;382(Pt 2):619–29. 10.1042/bj20040139. 15193147 10.1042/BJ20040139 PMC1133819

[137] Cazzolli R, Carpenter L, Biden TJ, Schmitz-Peiffer C. A role for protein phosphatase 2A–like activity, but not atypical protein kinase Cζ, in the inhibition of protein kinase B/Akt and glycogen synthesis by palmitate. Diabetes. 2001;50(10):2210–8. 10.2337/diabetes.50.10.2210. 11574400 10.2337/diabetes.50.10.2210

[138] Hassan RH, de Sousa ACP, Mahfouz R, Hainault I, Blachnio-Zabielska A, Bourron O, et al. Sustained action of ceramide on the insulin signaling pathway in muscle cells: implication of the double-stranded RNA-activated protein kinase. J Biol Chem. 2016;291(6):3019–29. 26698173 10.1074/jbc.M115.686949 PMC4742763

[139] Pellegrinelli V, Rouault C, Rodriguez-Cuenca S, Albert V, Edom-Vovard F, Vidal-Puig A, et al. Human adipocytes induce inflammation and atrophy in muscle cells during obesity. Diabetes. 2015;64(9):3121–34. 25695947 10.2337/db14-0796

[140] Chen Z-T, Weng Z-X, Lin JD, Meng Z-X. Myokines: metabolic regulation in obesity and type 2 diabetes. Life Metab. 2024;3(3):loae006. 10.1093/lifemeta/loae006. 39872377 10.1093/lifemeta/loae006 PMC11749576

[141] Togashi N, Ura N, Higashiura K, Murakami H, Shimamoto K. The contribution of skeletal muscle tumor necrosis factor-α to insulin resistance and hypertension in fructose-fed rats. J Hypertens. 2000;18(11):1605–10. 10.1097/00004872-200018110-00011. 11081773 10.1097/00004872-200018110-00011

[142] Uysal KT, Wiesbrock SM, Marino MW, Hotamisligil GS. Protection from obesity-induced insulin resistance in mice lacking TNF-α function. Nature. 1997;389(6651):610–4. 10.1038/39335. 9335502 10.1038/39335

[143] Fritsche L, Neukamm SS, Lehmann R, Kremmer E, Hennige AM, Hunder-Gugel A, et al. Insulin-induced serine phosphorylation of IRS-2 via ERK1/2 and mTOR: studies on the function of Ser675 and Ser907. Am J Physiol-Endocrinol Metab. 2011;300(5):E824–36. 10.1152/ajpendo.00409.2010. 21098738 10.1152/ajpendo.00409.2010

[144] Solinas G, Karin M. JNK1 and IKKβ: molecular links between obesity and metabolic dysfunction. FASEB J. 2010;24(8):2596–611. 20371626 10.1096/fj.09-151340

[145] Thoma A, Lightfoot AP. NF-kB and inflammatory cytokine signalling: role in skeletal muscle atrophy. Muscle Atrophy. 2018. 267–79. 10.1007/978-981-13-1435-3_12. 10.1007/978-981-13-1435-3_12 30390256

[146] Plomgaard P, Bouzakri K, Krogh-Madsen R, Mittendorfer B, Zierath JR, Pedersen BK. Tumor necrosis factor-α induces skeletal muscle insulin resistance in healthy human subjects via inhibition of Akt substrate 160 phosphorylation. Diabetes. 2005;54(10):2939–45. 10.2337/diabetes.54.10.2939. 16186396 10.2337/diabetes.54.10.2939

[147] de Alvaro C, Teruel T, Hernandez R, Lorenzo M. Tumor necrosis factor alpha produces insulin resistance in skeletal muscle by activation of inhibitor kappaB kinase in a p38 MAPK-dependent manner. J Biol Chem. 2004;279(17):17070–8. 10.1074/jbc.M312021200. 14764603 10.1074/jbc.M312021200

[148] Li P, Oh DY, Bandyopadhyay G, Lagakos WS, Talukdar S, Osborn O, et al. LTB4 promotes insulin resistance in obese mice by acting on macrophages, hepatocytes and myocytes. Nat Med. 2015;21(3):239–47. 10.1038/nm.3800. 25706874 10.1038/nm.3800 PMC4429798

[149] Luo G, Hershko DD, Robb BW, Wray CJ, Hasselgren P-O. IL-1β stimulates IL-6 production in cultured skeletal muscle cells through activation of MAP kinase signaling pathway and NF-κB. Am J Physiol-Regul Integr Comp Physiol. 2003;284(5):R1249–54. 10.1152/ajpregu.00490.2002. 12676746 10.1152/ajpregu.00490.2002

[150] Ghanbari M, Momen Maragheh S, Aghazadeh A, Mehrjuyan SR, Hussen BM, Abdoli Shadbad M, et al. Interleukin-1 in obesity-related low-grade inflammation: From molecular mechanisms to therapeutic strategies. Int Immunopharmacol. 2021;96:107765. 10.1016/j.intimp.2021.107765. 34015596 10.1016/j.intimp.2021.107765

[151] Gao D, Madi M, Ding C, Fok M, Steele T, Ford C, et al. Interleukin-1β mediates macrophage-induced impairment of insulin signaling in human primary adipocytes. Am J Physiol-Endocrinol Metab. 2014;307(3):E289–304. 10.1152/ajpendo.00430.2013. 24918199 10.1152/ajpendo.00430.2013 PMC4121578

[152] Larsen CM, Faulenbach M, Vaag A, Vølund A, Ehses JA, Seifert B, et al. Interleukin-1–receptor antagonist in type 2 diabetes mellitus. N Engl J Med. 2007;356(15):1517–26. 17429083 10.1056/NEJMoa065213

[153] Shi H, Kokoeva MV, Inouye K, Tzameli I, Yin H, Flier JS. TLR4 links innate immunity and fatty acid–induced insulin resistance. J Clin Investig. 2006;116(11):3015–25. 17053832 10.1172/JCI28898 PMC1616196

[154] Lee JY, Plakidas A, Lee WH, Heikkinen A, Chanmugam P, Bray G, et al. Differential modulation of Toll-like receptors by fatty acids: preferential inhibition by n-3 polyunsaturated fatty acids. J Lipid Res. 2003;44(3):479–86. 12562875 10.1194/jlr.M200361-JLR200

[155] Hwang DH, Kim J-A, Lee JY. Mechanisms for the activation of Toll-like receptor 2/4 by saturated fatty acids and inhibition by docosahexaenoic acid. Eur J Pharmacol. 2016;785:24–35. 10.1016/j.ejphar.2016.04.024. 27085899 10.1016/j.ejphar.2016.04.024 PMC5815395

[156] Wen H, Gris D, Lei Y, Jha S, Zhang L, Huang MT-H, et al. Fatty acid–induced NLRP3-ASC inflammasome activation interferes with insulin signaling. Nat Immunol. 2011;12(5):408–15. 10.1038/ni.2022. 21478880 10.1038/ni.2022 PMC4090391

[157] Russell-Guzmán J, Américo-Da Silva L, Cadagan C, Maturana M, Palomero J, Estrada M, et al. Activation of the ROS/TXNIP/NLRP3 pathway disrupts insulin-dependent glucose uptake in skeletal muscle of insulin-resistant obese mice. Free Radical Biol Med. 2024;222:187–98. 10.1016/j.freeradbiomed.2024.06.011. 38897422 10.1016/j.freeradbiomed.2024.06.011

[158] Américo-Da-Silva L, Aguilera J, Quinteros-Waltemath O, Sánchez-Aguilera P, Russell J, Cadagan C, et al. Activation of the NLRP3 inflammasome increases the IL-1β level and decreases GLUT4 translocation in skeletal muscle during insulin resistance. Int J Mol Sci. 2021;22(19):10212. 34638553 10.3390/ijms221910212 PMC8508423

[159] Eggelbusch M, Shi A, Broeksma BC, Vázquez-Cruz M, Soares MN, de Wit GMJ, et al. The NLRP3 inflammasome contributes to inflammation-induced morphological and metabolic alterations in skeletal muscle. J Cachexia Sarcopenia Muscle. 2022;13(6):3048–61. 10.1002/jcsm.13062. 35978267 10.1002/jcsm.13062 PMC9745466

[160] Grzelkowska-Kowalczyk K, Wieteska-Skrzeczyńska W. Treatment with IFN-γ prevents insulin-dependent PKB, p70S6k phosphorylation and protein synthesis in mouse C2C12 myogenic cells. Cell Biol Int. 2010;34(1):117–24. 10.1042/CBI20090135. 10.1042/CBI20090135 19947939

[161] Ivashkiv LB. IFNγ: signalling, epigenetics and roles in immunity, metabolism, disease and cancer immunotherapy. Nat Rev Immunol. 2018;18(9):545–58. 29921905 10.1038/s41577-018-0029-z PMC6340644

[162] Zhang L, Chen Z, Wang Y, Tweardy DJ, Mitch WE. Stat3 activation induces insulin resistance via a muscle-specific E3 ubiquitin ligase Fbxo40. Am J Physiol Endocrinol Metab. 2020;318(5):E625–35. 10.1152/ajpendo.00480.2019. 32101031 10.1152/ajpendo.00480.2019 PMC7272729

[163] Abid H, Ryan ZC, Delmotte P, Sieck GC, Lanza IR. Extramyocellular interleukin-6 influences skeletal muscle mitochondrial physiology through canonical JAK/STAT signaling pathways. FASEB J. 2020;34(11):14458–72. 10.1096/fj.202000965RR. 32885495 10.1096/fj.202000965RR

[164] Wu CL, Kandarian SC, Jackman RW. Identification of genes that elicit disuse muscle atrophy via the transcription factors p50 and Bcl-3. PLoS One. 2011;6(1):e16171. 10.1371/journal.pone.0016171. 21249144 10.1371/journal.pone.0016171 PMC3020958

[165] Cai D, Frantz JD, Tawa NE, Melendez PA, Oh B-C, Lidov HGW, et al. IKKβ/NF-κB activation causes severe muscle wasting in mice. Cell. 2004;119(2):285–98. 10.1016/j.cell.2004.09.027. 15479644 10.1016/j.cell.2004.09.027

[166] Li YP, Chen Y, John J, Moylan J, Jin B, Mann DL, et al. TNF-alpha acts via p38 MAPK to stimulate expression of the ubiquitin ligase atrogin1/MAFbx in skeletal muscle. FASEB J : Off Publ Fed Am Soc Exp Biol. 2005;19(3):362–70. 10.1096/fj.04-2364com. 10.1096/fj.04-2364com PMC3099533 15746179

[167] Bonetto A, Aydogdu T, Jin X, Zhang Z, Zhan R, Puzis L, et al. JAK/STAT3 pathway inhibition blocks skeletal muscle wasting downstream of IL-6 and in experimental cancer cachexia. Am J Physiol-Endocrinol Metab. 2012;303(3):E410–21. 10.1152/ajpendo.00039.2012. 22669242 10.1152/ajpendo.00039.2012 PMC3423125

[168] Zhang L, Pan J, Dong Y, Tweardy DJ, Dong Y, Garibotto G, et al. Stat3 activation links a C/EBPδ to myostatin pathway to stimulate loss of muscle mass. Cell Metab. 2013;18(3):368–79. 10.1016/j.cmet.2013.07.012. 24011072 10.1016/j.cmet.2013.07.012 PMC3794464

[169] Chomentowski P, Coen PM, Radiková Z, Goodpaster BH, Toledo FG. Skeletal muscle mitochondria in insulin resistance: differences in intermyofibrillar versus subsarcolemmal subpopulations and relationship to metabolic flexibility. J Clin Endocrinol Metab. 2011;96(2):494–503. 10.1210/jc.2010-0822. 21106709 10.1210/jc.2010-0822 PMC3048328

[170] Patti ME, Butte AJ, Crunkhorn S, Cusi K, Berria R, Kashyap S, et al. Coordinated reduction of genes of oxidative metabolism in humans with insulin resistance and diabetes: Potential role of PGC1 and NRF1. Proc Natl Acad Sci USA. 2003;100(14):8466–71. 10.1073/pnas.1032913100. 12832613 10.1073/pnas.1032913100 PMC166252

[171] Heilbronn LK, Gan SK, Turner N, Campbell LV, Chisholm DJ. Markers of mitochondrial biogenesis and metabolism are lower in overweight and obese insulin-resistant subjects. J Clin Endocrinol Metab. 2007;92(4):1467–73. 10.1210/jc.2006-2210. 17244782 10.1210/jc.2006-2210

[172] Russell AP, Feilchenfeldt J, Schreiber S, Praz M, Crettenand A, Gobelet C, et al. Endurance training in humans leads to fiber type-specific increases in levels of peroxisome proliferator-activated receptor-gamma coactivator-1 and peroxisome proliferator-activated receptor-alpha in skeletal muscle. Diabetes. 2003;52(12):2874–81. 10.2337/diabetes.52.12.2874. 14633846 10.2337/diabetes.52.12.2874

[173] Boyle KE, Canham JP, Consitt LA, Zheng D, Koves TR, Gavin TP, et al. A high-fat diet elicits differential responses in genes coordinating oxidative metabolism in skeletal muscle of lean and obese individuals. J Clin Endocrinol Metab. 2011;96(3):775–81. 10.1210/jc.2010-2253. 21190973 10.1210/jc.2010-2253 PMC3047224

[174] Richardson DK, Kashyap S, Bajaj M, Cusi K, Mandarino SJ, Finlayson J, et al. Lipid infusion decreases the expression of nuclear encoded mitochondrial genes and increases the expression of extracellular matrix genes in human skeletal muscle. J Biol Chem. 2005;280(11):10290–7. 10.1074/jbc.M408985200. 15598661 10.1074/jbc.M408985200

[175] Kong S, Cai B, Nie Q. PGC-1α affects skeletal muscle and adipose tissue development by regulating mitochondrial biogenesis. Mol Genet Genomics. 2022;297(3):621–33. 10.1007/s00438-022-01878-2. 35290519 10.1007/s00438-022-01878-2

[176] Figueroa-Toledo AM, Gutiérrez-Pino J, Carriel-Nesvara A, Marchese-Bittencourt M, Zbinden-Foncea H, Castro-Sepúlveda M. BMAL1 and CLOCK proteins exhibit differential association with mitochondrial dynamics, protein synthesis pathways and muscle strength in human muscle. J Physiol. 2024. 10.1113/jp285955. 38922907 10.1113/JP285955

[177] Wefers J, Connell NJ, Fealy CE, Andriessen C, de Wit V, van Moorsel D, et al. Day-night rhythm of skeletal muscle metabolism is disturbed in older, metabolically compromised individuals. Mol Metab. 2020;41:101050. 10.1016/j.molmet.2020.101050. 32659272 10.1016/j.molmet.2020.101050 PMC7415921

[178] Houzelle A, Jörgensen JA, Schaart G, Daemen S, van Polanen N, Fealy CE, et al. Human skeletal muscle mitochondrial dynamics in relation to oxidative capacity and insulin sensitivity. Diabetologia. 2021;64(2):424–36. 10.1007/s00125-020-05335-w. 33258025 10.1007/s00125-020-05335-w PMC7801361

[179] Gemmink A, Daemen S, Wefers J, Hansen J, van Moorsel D, Astuti P, et al. Twenty-four hour rhythmicity in mitochondrial network connectivity and mitochondrial respiration; a study in human skeletal muscle biopsies of young lean and older individuals with obesity. Mol Metab. 2023;72:101727. 10.1016/j.molmet.2023.101727. 37062525 10.1016/j.molmet.2023.101727 PMC10160594

[180] Gabriel BM, Altıntaş A, Smith JAB, Sardon-Puig L, Zhang X, Basse AL, et al. Disrupted circadian oscillations in type 2 diabetes are linked to altered rhythmic mitochondrial metabolism in skeletal muscle. Sci Adv. 2021;7(43):eabi9654. 10.1126/sciadv.abi9654. 34669477 10.1126/sciadv.abi9654 PMC8528429

[181] Liu R, Jin P, Yu L, Wang Y, Han L, Shi T, et al. Impaired mitochondrial dynamics and bioenergetics in diabetic skeletal muscle. PloS one. 2014;9(3):e92810. 24658162 10.1371/journal.pone.0092810 PMC3962456

[182] Jheng H-F, Tsai P-J, Guo S-M, Kuo L-H, Chang C-S, Su I-J, et al. Mitochondrial fission contributes to mitochondrial dysfunction and insulin resistance in skeletal muscle. Mol Cell Biol. 2012;32(2):309–19. 10.1128/MCB.05603-11. 22083962 10.1128/MCB.05603-11 PMC3255771

[183] Dai W, Jiang L. Dysregulated mitochondrial dynamics and metabolism in obesity, diabetes, and cancer. Front Endocrinol. 2019;10:570. 10.3389/fendo.2019.00570 PMC6734166 31551926

[184] Kugler BA, Labaf M, Nguyen P, Lin N, Houmard JA, Zarringhalam K, et al. The loss of Drp1 improves skeletal muscle insulin action in primary myotubes derived from humans with severe obesity. FASEB J. 2022;36(S1):R4873. 10.1096/fasebj.2022.36.S1.R4873.

[185] Axelrod CL, Fealy CE, Erickson ML, Davuluri G, Fujioka H, Dantas WS, et al. Lipids activate skeletal muscle mitochondrial fission and quality control networks to induce insulin resistance in humans. Metabolism. 2021;121:154803. 34090870 10.1016/j.metabol.2021.154803 PMC8277749

[186] Smith ME, Tippetts TS, Brassfield ES, Tucker BJ, Ockey A, Swensen AC, et al. Mitochondrial fission mediates ceramide-induced metabolic disruption in skeletal muscle. Biochem J. 2013;456(3):427–39. 24073738 10.1042/BJ20130807

[187] Bikman BT. Mitochondrial fission is necessary for ceramide-induced metabolic disruption. FASEB J. 2016;30(S1):307.5-.5. 10.1096/fasebj.30.1_supplement.307.5.

[188] Perreault L, Newsom SA, Strauss A, Kerege A, Kahn DE, Harrison KA, et al. Intracellular localization of diacylglycerols and sphingolipids influences insulin sensitivity and mitochondrial function in human skeletal muscle. JCI Insight. 2018;3(3):e96805. 10.1172/jci.insight.96805. 29415895 10.1172/jci.insight.96805 PMC5821197

[189] Diaz-Vegas A, Madsen S, Cooke KC, Carroll L, Khor JX, Turner N, et al. Mitochondrial electron transport chain, ceramide, and coenzyme Q are linked in a pathway that drives insulin resistance in skeletal muscle. Elife. 2023;12:RP87340. 38149844 10.7554/eLife.87340 PMC10752590

[190] Finocchietto P, Perez H, Blanco G, Miksztowicz V, Marotte C, Morales C, et al. Inhibition of mitochondrial fission by Drp-1 blockade by short-term leptin and Mdivi-1 treatment improves white adipose tissue abnormalities in obesity and diabetes. Pharmacol Res. 2022;178:106028. 10.1016/j.phrs.2021.106028. 34896541 10.1016/j.phrs.2021.106028

[191] Liesa M, Palacín M, Zorzano A. Mitochondrial dynamics in mammalian health and disease. Physiol Rev. 2009;89(3):799–845. 19584314 10.1152/physrev.00030.2008

[192] Bach D, Pich S, Soriano FX, Vega N, Baumgartner B, Oriola J, et al. Mitofusin-2 determines mitochondrial network architecture and mitochondrial metabolism. A novel regulatory mechanism altered in obesity. J Biol Chem. 2003;278(19):17190–7. 10.1074/jbc.M212754200. 12598526 10.1074/jbc.M212754200

[193] Bach D, Naon D, Pich S, Soriano FX, Vega N, Rieusset J, et al. Expression of Mfn2, the Charcot-Marie-Tooth neuropathy type 2A gene, in human skeletal muscle: effects of type 2 diabetes, obesity, weight loss, and the regulatory role of tumor necrosis factor alpha and interleukin-6. Diabetes. 2005;54(9):2685–93. 10.2337/diabetes.54.9.2685. 16123358 10.2337/diabetes.54.9.2685

[194] Fealy CE, Mulya A, Axelrod CL, Kirwan JP. Mitochondrial dynamics in skeletal muscle insulin resistance and type 2 diabetes. Transl Res. 2018;202:69–82. 30153426 10.1016/j.trsl.2018.07.011

[195] Pereira RO, Tadinada SM, Zasadny FM, Oliveira KJ, Pires KMP, Olvera A, et al. OPA1 deficiency promotes secretion of FGF21 from muscle that prevents obesity and insulin resistance. EMBO J. 2017;36(14):2126–45. 10.15252/embj.201696179. 28607005 10.15252/embj.201696179 PMC5510002

[196] Favaro G, Romanello V, Varanita T, Andrea Desbats M, Morbidoni V, Tezze C, et al. DRP1-mediated mitochondrial shape controls calcium homeostasis and muscle mass. Nat Commun. 2019;10(1):2576. 10.1038/s41467-019-10226-9. 31189900 10.1038/s41467-019-10226-9 PMC6561930

[197] Nisr RB, Shah DS, Ganley IG, Hundal HS. Proinflammatory NFkB signalling promotes mitochondrial dysfunction in skeletal muscle in response to cellular fuel overloading. Cell Mol Life Sci. 2019;76:4887–904. 31101940 10.1007/s00018-019-03148-8 PMC6881256

[198] Di Meo S, Iossa S, Venditti P. Skeletal muscle insulin resistance: role of mitochondria and other ROS sources. J Endocrinol. 2017;233(1):R15–42. 28232636 10.1530/JOE-16-0598

[199] Lin H-Y, Weng S-W, Chang Y-H, Su Y-J, Chang C-M, Tsai C-J, et al. The causal role of mitochondrial dynamics in regulating insulin resistance in diabetes: link through mitochondrial reactive oxygen species. Oxid Med Cell Longev. 2018;2018(1):7514383. 10.1155/2018/7514383. 30363990 10.1155/2018/7514383 PMC6186363

[200] Fan X, Hussien R, Brooks GA. H2O2-induced mitochondrial fragmentation in C2C12 myocytes. Free Radical Biol Med. 2010;49(11):1646–54. 10.1016/j.freeradbiomed.2010.08.024. 20801212 10.1016/j.freeradbiomed.2010.08.024 PMC2970628

[201] Anderson EJ, Lustig ME, Boyle KE, Woodlief TL, Kane DA, Lin CT, et al. Mitochondrial H2O2 emission and cellular redox state link excess fat intake to insulin resistance in both rodents and humans. J Clin Invest. 2009;119(3):573–81. 10.1172/jci37048. 19188683 10.1172/JCI37048 PMC2648700

[202] Lark DS, Kang L, Lustig ME, Bonner JS, James FD, Neufer PD, et al. Enhanced mitochondrial superoxide scavenging does not improve muscle insulin action in the high fat-fed mouse. PLoS One. 2015;10(5):e0126732. 10.1371/journal.pone.0126732. 25992608 10.1371/journal.pone.0126732 PMC4437982

[203] Houstis N, Rosen ED, Lander ES. Reactive oxygen species have a causal role in multiple forms of insulin resistance. Nature. 2006;440(7086):944–8. 10.1038/nature04634. 16612386 10.1038/nature04634

[204] Chen L, Na R, Gu M, Salmon AB, Liu Y, Liang H, et al. Reduction of mitochondrial H2O2 by overexpressing peroxiredoxin 3 improves glucose tolerance in mice. Aging Cell. 2008;7(6):866–78. 10.1111/j.1474-9726.2008.00432.x. 18778410 10.1111/j.1474-9726.2008.00432.x PMC4431549

[205] Boden MJ, Brandon AE, Tid-Ang JD, Preston E, Wilks D, Stuart E, et al. Overexpression of manganese superoxide dismutase ameliorates high-fat diet-induced insulin resistance in rat skeletal muscle. Am J Physiol Endocrinol Metab. 2012;303(6):E798-805. 10.1152/ajpendo.00577.2011. 22829583 10.1152/ajpendo.00577.2011 PMC3468429

[206] Aguirre V, Uchida T, Yenush L, Davis R, White MF. The c-Jun NH2-terminal kinase promotes insulin resistance during association with insulin receptor substrate-1 and phosphorylation of Ser307. J Biol Chem. 2000;275(12):9047–54. 10722755 10.1074/jbc.275.12.9047

[207] Tuncman G, Hirosumi J, Solinas G, Chang L, Karin M, Hotamisligil GS. Functional *in vivo* interactions between JNK1 and JNK2 isoforms in obesity and insulin resistance. Proc Natl Acad Sci USA. 2006;103(28):10741–6. 10.1073/pnas.0603509103. 16818881 10.1073/pnas.0603509103 PMC1487172

[208] Fujishiro M, Gotoh Y, Katagiri H, Sakoda H, Ogihara T, Anai M, et al. MKK6/3 and p38 MAPK pathway activation is not necessary for insulin-induced glucose uptake but regulates glucose transporter expression. J Biol Chem. 2001;276(23):19800–6. 11279172 10.1074/jbc.M101087200

[209] Archuleta TL, Lemieux AM, Saengsirisuwan V, Teachey MK, Lindborg KA, Kim JS, et al. Oxidant stress-induced loss of IRS-1 and IRS-2 proteins in rat skeletal muscle: Role of p38 MAPK. Free Radical Biol Med. 2009;47(10):1486–93. 10.1016/j.freeradbiomed.2009.08.014. 19703555 10.1016/j.freeradbiomed.2009.08.014 PMC2767452

[210] Wei Y, Sowers JR, Nistala R, Gong H, Uptergrove GM-E, Clark SE, et al. Angiotensin II-induced NADPH oxidase activation impairs insulin signaling in skeletal muscle cells. J Biol Chem. 2006;281(46):35137–46. 16982630 10.1074/jbc.M601320200

[211] Wei Y, Sowers JR, Clark SE, Li W, Ferrario CM, Stump CS. Angiotensin II-induced skeletal muscle insulin resistance mediated by NF-κB activation via NADPH oxidase. Am J Physiol-Endocrinol Metab. 2008;294(2):E345–51. 10.1152/ajpendo.00456.2007. 18073321 10.1152/ajpendo.00456.2007

[212] Pillon NJ, Croze ML, Vella RE, Soulère L, Lagarde M, Soulage CO. The lipid peroxidation by-product 4-hydroxy-2-nonenal (4-HNE) induces insulin resistance in skeletal muscle through both carbonyl and oxidative stress. Endocrinology. 2012;153(5):2099–111. 10.1210/en.2011-1957. 22396448 10.1210/en.2011-1957

[213] Lee H, Ha TY, Jung CH, Nirmala FS, Park S-Y, Huh YH, et al. Mitochondrial dysfunction in skeletal muscle contributes to the development of acute insulin resistance in mice. J Cachexia Sarcopenia Muscle. 2021;12(6):1925–39. 10.1002/jcsm.12794. 34605225 10.1002/jcsm.12794 PMC8718067

[214] Tsilingiris D, Tzeravini E, Koliaki C, Dalamaga M, Kokkinos A. The role of mitochondrial adaptation and metabolic flexibility in the pathophysiology of obesity and insulin resistance: an updated overview. Curr Obes Rep. 2021;10(3):191–213. 10.1007/s13679-021-00434-0. 33840072 10.1007/s13679-021-00434-0

[215] Schiaffino S. Fibre types in skeletal muscle: a personal account. Acta Physiol (Oxf). 2010;199(4):451–63. 10.1111/j.1748-1716.2010.02130.x. 20353491 10.1111/j.1748-1716.2010.02130.x

[216] Stuart CA, McCurry MP, Marino A, South MA, Howell MEA, Layne AS, et al. Slow-twitch fiber proportion in skeletal muscle correlates with insulin responsiveness. J Clin Endocrinol Metab. 2013;98(5):2027–36. 10.1210/jc.2012-3876. 23515448 10.1210/jc.2012-3876 PMC3644602

[217] Albers PH, Pedersen AJT, Birk JB, Kristensen DE, Vind BF, Baba O, et al. Human muscle fiber type-specific insulin signaling: impact of obesity and type 2 diabetes. Diabetes. 2014;64(2):485–97. 10.2337/db14-0590. 25187364 10.2337/db14-0590

[218] Daugaard JR, Nielsen JN, Kristiansen S, Andersen JL, Hargreaves M, Richter EA. Fiber type-specific expression of GLUT4 in human skeletal muscle: influence of exercise training. Diabetes. 2000;49(7):1092–5. 10.2337/diabetes.49.7.1092. 10909963 10.2337/diabetes.49.7.1092

[219] Schiaffino S, Reggiani C. Fiber types in mammalian skeletal muscles. Physiol Rev. 2011;91(4):1447–531. 10.1152/physrev.00031.2010. 22013216 10.1152/physrev.00031.2010

[220] Damer A, El Meniawy S, McPherson R, Wells G, Harper ME, Dent R. Association of muscle fiber type with measures of obesity: A systematic review. Obes Rev : Off J Int Assoc Study Obes. 2022;23(7):e13444. 10.1111/obr.13444. 10.1111/obr.13444 35293095

[221] Lin J, Wu H, Tarr PT, Zhang C-Y, Wu Z, Boss O, et al. Transcriptional co-activator PGC-1α drives the formation of slow-twitch muscle fibres. Nature. 2002;418(6899):797–801. 12181572 10.1038/nature00904

[222] Handschin C, Rhee J, Lin J, Tarr PT, Spiegelman BM. An autoregulatory loop controls peroxisome proliferator-activated receptor gamma coactivator 1alpha expression in muscle. Proc Natl Acad Sci USA. 2003;100(12):7111–6. 10.1073/pnas.1232352100. 12764228 10.1073/pnas.1232352100 PMC165838

[223] Luquet S, Lopez-Soriano J, Holst D, Fredenrich A, Melki J, Rassoulzadegan M, et al. Peroxisome proliferator-activated receptor δ controls muscle development and oxydative capability. FASEB J. 2003;17(15):2299–301. 10.1096/fj.03-0269fje. 14525942 10.1096/fj.03-0269fje

[224] Wang Y-X, Zhang C-L, Yu RT, Cho HK, Nelson MC, Bayuga-Ocampo CR, et al. Regulation of muscle fiber type and running endurance by PPARδ. PLoS Biol. 2004;2(10):e294. 10.1371/journal.pbio.0020294. 15328533 10.1371/journal.pbio.0020294 PMC509410

[225] Iwabu M, Yamauchi T, Okada-Iwabu M, Sato K, Nakagawa T, Funata M, et al. Adiponectin and AdipoR1 regulate PGC-1α and mitochondria by Ca2+ and AMPK/SIRT1. Nature. 2010;464(7293):1313–9. 20357764 10.1038/nature08991

[226] Zhou Q, Gu Y, Lang H, Wang X, Chen K, Gong X, et al. Dihydromyricetin prevents obesity-induced slow-twitch-fiber reduction partially via FLCN/FNIP1/AMPK pathway. Biochim Biophys Acta. 2017;1863(6):1282–91. 10.1016/j.bbadis.2017.03.019. 10.1016/j.bbadis.2017.03.019 28363698

[227] Hu M-M, Zheng W-Y, Cheng M-H, Song Z-Y, Shaukat H, Atta M, et al. Sesamol reverses myofiber-type conversion in obese states via activating the SIRT1/AMPK signal pathway. J Agric Food Chem. 2022;70(7):2253–64. 35166533 10.1021/acs.jafc.1c08036

[228] Jiang Q, Cheng X, Cui Y, Xia Q, Yan X, Zhang M, et al. Resveratrol regulates skeletal muscle fibers switching through the AdipoR1-AMPK-PGC-1α pathway. Food Funct. 2019;10(6):3334–43. 31095141 10.1039/c8fo02518e

[229] Chin ER, Olson EN, Richardson JA, Yang Q, Humphries C, Shelton JM, et al. A calcineurin-dependent transcriptional pathway controls skeletal muscle fiber type. Genes Dev. 1998;12(16):2499–509. 9716403 10.1101/gad.12.16.2499 PMC317085

[230] Eshima H. Influence of obesity and type 2 diabetes on calcium handling by skeletal muscle: spotlight on the sarcoplasmic reticulum and mitochondria. Front Physiol. 2021;12:758316. 34795598 10.3389/fphys.2021.758316 PMC8592904

[231] Bottinelli R, Canepari M, Pellegrino MA, Reggiani C. Force-velocity properties of human skeletal muscle fibres: myosin heavy chain isoform and temperature dependence. J Physiol. 1996;495((Pt 2)(Pt 2)):573–86. 10.1113/jphysiol.1996.sp021617. 8887767 10.1113/jphysiol.1996.sp021617 PMC1160815

[232] Straight CR, Toth MJ, Miller MS. Current perspectives on obesity and skeletal muscle contractile function in older adults. J Appl Physiol (Bethesda, Md : 1985). 2021;130(1):10–6. 10.1152/japplphysiol.00739.2020. 10.1152/japplphysiol.00739.2020 PMC7944932 33211593

[233] Twarda-Clapa A, Olczak A, Białkowska AM, Koziołkiewicz M. Advanced glycation end-products (AGEs): Formation, chemistry, classification, receptors, and diseases related to AGEs. Cells. 2022;11(8):1312. 35455991 10.3390/cells11081312 PMC9029922

[234] Khalid M, Petroianu G, Adem A. Advanced glycation end products and diabetes mellitus: mechanisms and perspectives. Biomolecules. 2022;12(4):542. 35454131 10.3390/biom12040542 PMC9030615

[235] Peeters SA, Engelen L, Buijs J, Theilade S, Rossing P, Schalkwijk CG, et al. Associations between advanced glycation endproducts and matrix metalloproteinases and its inhibitor in individuals with type 1 diabetes. J Diabetes Complicat. 2018;32(3):325–9. 10.1016/j.jdiacomp.2017.12.011. 10.1016/j.jdiacomp.2017.12.011 29395841

[236] Egawa T, Tsuda S, Goto A, Ohno Y, Yokoyama S, Goto K, et al. Potential involvement of dietary advanced glycation end products in impairment of skeletal muscle growth and muscle contractile function in mice. Br J Nutr. 2017;117(1):21–9. 10.1017/S0007114516004591. 28093090 10.1017/S0007114516004591

[237] Ahmad K, Lee EJ, Moon JS, Park S-Y, Choi I. Multifaceted interweaving between extracellular matrix, insulin resistance, and skeletal muscle. Cells. 2018;7(10):148. 30249008 10.3390/cells7100148 PMC6211053

[238] Riuzzi F, Sorci G, Sagheddu R, Chiappalupi S, Salvadori L, Donato R. RAGE in the pathophysiology of skeletal muscle. J Cachexia Sarcopenia Muscle. 2018;9(7):1213–34. 30334619 10.1002/jcsm.12350 PMC6351676

[239] Miranda ER, Mey JT, Blackburn BK, Chaves AB, Fuller KNZ, Perkins RK, et al. Soluble RAGE and skeletal muscle tissue RAGE expression profiles in lean and obese young adults across differential aerobic exercise intensities. J Appl Physiol (Bethesda, Md : 1985). 2023;135(4):849–62. 10.1152/japplphysiol.00748.2022. 10.1152/japplphysiol.00748.2022 PMC10642519 37675469

[240] Chiu CY, Yang RS, Sheu ML, Chan DC, Yang TH, Tsai KS, et al. Advanced glycation end-products induce skeletal muscle atrophy and dysfunction in diabetic mice via a RAGE-mediated, AMPK-down-regulated, Akt pathway. J Pathol. 2016;238(3):470–82. 26586640 10.1002/path.4674

[241] Wautier M-P, Chappey O, Corda S, Stern DM, Schmidt AM, Wautier J-L. Activation of NADPH oxidase by AGE links oxidant stress to altered gene expression via RAGE. Am J Physiol-Endocrinol Metab. 2001;280(5):E685–94. 10.1152/ajpendo.2001.280.5.E685. 11287350 10.1152/ajpendo.2001.280.5.E685

[242] Coughlan MT, Thorburn DR, Penfold SA, Laskowski A, Harcourt BE, Sourris KC, et al. RAGE-induced cytosolic ROS promote mitochondrial superoxide generation in diabetes. J Am Soc Nephrol. 2009;20(4):742–52. 10.1681/asn.2008050514. 19158353 10.1681/ASN.2008050514 PMC2663823

[243] Egawa T, Ogawa T, Yokokawa T, Kido K, Iyama R, Zhao H, et al. Glycative stress inhibits hypertrophy and impairs cell membrane integrity in overloaded mouse skeletal muscle. J Cachexia Sarcopenia Muscle. 2024;15(3):883–96. 10.1002/jcsm.13444. 38575520 10.1002/jcsm.13444 PMC11154761

[244] Du H, Ma Y, Wang X, Zhang Y, Zhu L, Shi S, et al. Advanced glycation end products induce skeletal muscle atrophy and insulin resistance via activating ROS-mediated ER stress PERK/FOXO1 signaling. Am J Physiol-Endocrinol Metab. 2023;324(3):E279–87. 36724125 10.1152/ajpendo.00218.2022

[245] Miranda ER, Somal VS, Mey JT, Blackburn BK, Wang E, Farabi S, et al. Circulating soluble RAGE isoforms are attenuated in obese, impaired-glucose-tolerant individuals and are associated with the development of type 2 diabetes. Am J Physiol-Endocrinol Metab. 2017;313(6):E631–40. 10.1152/ajpendo.00146.2017. 28811295 10.1152/ajpendo.00146.2017 PMC5814601

[246] Cid-Díaz T, Leal-López S, Fernández-Barreiro F, González-Sánchez J, Santos-Zas I, Andrade-Bulos LJ, et al. Obestatin signalling counteracts glucocorticoid-induced skeletal muscle atrophy via NEDD4/KLF15 axis. J Cachexia Sarcopenia Muscle. 2021;12(2):493–505. 10.1002/jcsm.12677. 33687156 10.1002/jcsm.12677 PMC8061369

[247] Lorenzo I, Serra-Prat M, Yébenes JC. The role of water homeostasis in muscle function and frailty: a review. Nutrients. 2019;11(8):1857. 31405072 10.3390/nu11081857 PMC6723611

[248] Magkos F, Hjorth MF, Astrup A. Diet and exercise in the prevention and treatment of type 2 diabetes mellitus. Nat Rev Endocrinol. 2020;16(10):545–55. 10.1038/s41574-020-0381-5. 32690918 10.1038/s41574-020-0381-5

[249] Cosentino F, Grant PJ, Aboyans V, Bailey CJ, Ceriello A, Delgado V, et al. 2019 ESC Guidelines on diabetes, pre-diabetes, and cardiovascular diseases developed in collaboration with the EASD: The Task Force for diabetes, pre-diabetes, and cardiovascular diseases of the European Society of Cardiology (ESC) and the European Association for the Study of Diabetes (EASD). Eur Heart J. 2019;41(2):255–323. 10.1093/eurheartj/ehz486. 10.1093/eurheartj/ehz486 31497854

[250] Kanaley JA, Colberg SR, Corcoran MH, Malin SK, Rodriguez NR, Crespo CJ, et al. Exercise/physical activity in individuals with type 2 diabetes: a consensus statement from the American College of Sports Medicine. Med Sci Sports Exerc. 2022;54(2):353–68. 10.1249/mss.0000000000002800. 35029593 10.1249/MSS.0000000000002800 PMC8802999

[251] Phillips JA. Dietary guidelines for Americans, 2020–2025. Work Health Saf. 2021;69(8):395. 10.1177/21650799211026980 34279148

[252] Thyfault JP, Bergouignan A. Exercise and metabolic health: beyond skeletal muscle. Diabetologia. 2020;63(8):1464–74. 10.1007/s00125-020-05177-6. 32529412 10.1007/s00125-020-05177-6 PMC7377236

[253] McGee SL, Hargreaves M. Exercise adaptations: molecular mechanisms and potential targets for therapeutic benefit. Nat Rev Endocrinol. 2020;16(9):495–505. 32632275 10.1038/s41574-020-0377-1

[254] Egan B, Sharples AP. Molecular responses to acute exercise and their relevance for adaptations in skeletal muscle to exercise training. Physiol Rev. 2023;103(3):2057–170. 36395350 10.1152/physrev.00054.2021

[255] Richter EA. Is GLUT4 translocation the answer to exercise-stimulated muscle glucose uptake? Am J Physiol-Endocrinol Metab. 2021;320(2):E240–3. 10.1152/ajpendo.00503.2020. 33166188 10.1152/ajpendo.00503.2020 PMC8260367

[256] Plotkin DL, Roberts MD, Haun CT, Schoenfeld BJ. Muscle fiber type transitions with exercise training: shifting perspectives. Sports. 2021;9(9):127. 34564332 10.3390/sports9090127 PMC8473039

[257] Safdar A, Little JP, Stokl AJ, Hettinga BP, Akhtar M, Tarnopolsky MA. Exercise increases mitochondrial PGC-1α content and promotes nuclear-mitochondrial cross-talk to coordinate mitochondrial biogenesis. J Biol Chem. 2011;286(12):10605–17. 21245132 10.1074/jbc.M110.211466 PMC3060512

[258] Wackerhage H, Schoenfeld BJ, Hamilton DL, Lehti M, Hulmi JJ. Stimuli and sensors that initiate skeletal muscle hypertrophy following resistance exercise. J Appl Physiol. 2019;126(1):30–43. 10.1152/japplphysiol.00685.2018. 30335577 10.1152/japplphysiol.00685.2018

[259] Kistner TM, Pedersen BK, Lieberman DE. Interleukin 6 as an energy allocator in muscle tissue. Nat Metab. 2022;4(2):170–9. 10.1038/s42255-022-00538-4. 35210610 10.1038/s42255-022-00538-4

[260] Pérez-Castillo IM, Sabag-Daigle A, López-Chicharro J, Mihic N, Rueda R, Bouzamondo H. The athlete gut microbiota: state of the art and practical guidance. Benefic Microbes. 2024;1(aop):1–30. 10.1163/18762891-bja00007 38659188

